# A comprehensive study on enhancing of the mechanical properties of steel fiber-reinforced concrete through nano-silica integration

**DOI:** 10.1038/s41598-023-47475-0

**Published:** 2023-11-16

**Authors:** Anbuchezian Ashokan, Silambarasan Rajendran, Ratchagaraja Dhairiyasamy

**Affiliations:** 1 Department of Civil Engineering, Annapoorana Engineering College, Salem, Tamil Nadu India; 2 Department of Mechanical Engineering, Annapoorana Engineering College, Salem, Tamil Nadu India; 3 Department of Mechanical Engineering, College of Engineering and Technology, Aksum University, Aksum, Ethiopia

**Keywords:** Nanoscale materials, Civil engineering

## Abstract

Steel fiber reinforced concrete (SFRC) offers improved toughness, crack resistance, and impact resistance. Nano-silica enhances the strength, durability, and workability of concrete. This study investigated the combined effect of nano-silica and steel microfibers, termed micro-concrete reinforced with steel fibers embedding nano-silica (MRFAIN), on the mechanical properties of concrete. The aim was to determine the influence of different percentages of nano-silica and steel microfibers on fresh state properties, mechanical strength, and mechanical performance of MRFAIN. MRFAIN mixtures were prepared with cement, sand, water, superplasticizer, varying dosages of nano-silica (0–2%), and steel microfibers (0–2% by volume). Mechanical properties evaluated at 28 days included compressive strength, flexural strength, modulus of elasticity, and fracture energy. Incorporating steel microfibers reduced workability but enhanced mechanical properties like strength and ductility. Nano-silica addition showed variable effects on compressive strength but increased tensile strength. Optimal nano-silica content was 1% and steel microfibers 2%, giving compressive strength 122.5 MPa, tensile strength 25.4 MPa, modulus of elasticity 42.7 GPa. Using nano-silica and steel, microfibers enhanced the mechanical performance of steel fiber-reinforced concrete. This shows potential for reducing construction waste and pollution. Further research can optimize the proportions of nano-silica and steel microfibers in MRFAIN.

## Introduction

Concrete is one of the most widely used construction materials in the world. It primarily comprises cement, water, and aggregates such as sand and gravel. Concrete’s versatility, affordability, and structural capabilities allow it to be used for various applications in buildings, infrastructure, and construction projects. Concrete’s properties and performance characteristics are critical for determining its suitability and ensuring concrete structures’ durability, load-bearing capacity, and service life. Key properties that influence the quality and usefulness of concrete include strength, stiffness, durability, shrinkage, creep, permeability, and thermal properties. Extensive research has been conducted to understand further the factors affecting concrete properties and develop innovative concrete mixtures and materials. Standards and codes provide specifications and guidelines for concrete to ensure quality control during construction. However, variability in concrete constituents, curing conditions, and testing methods pose challenges. Ongoing advances in concrete technology aim to optimize properties and performance to expand concrete’s sustainable implementation in structural engineering^1,2^.

Some critical properties of concrete include strength, durability, workability, and shrinkage. Strength refers to the ability of concrete to resist forces and load, which is crucial for the stability and safety of structures. Durability refers to the ability of concrete to resist various environmental factors, such as weathering, chemical attacks, and physical impacts, and to maintain its strength and function over time^3^. Workability refers to the ease of mixing, placing, and finishing concrete, which is essential for achieving concrete’s desired consistency and quality. Shrinkage refers to the reduction in the volume of concrete due to drying and curing, which can cause cracking and other problems if not properly managed^4^. In addition to these properties, concrete can also be evaluated based on its thermal properties, sound insulation properties, and fire resistance, among others. The properties of concrete are influenced by several factors, including the composition of the concrete mixture, the curing conditions, the environment, and the loading and exposure conditions^5^.

Recent studies have shown that adding nano-silica can enhance the properties of conventional concrete by improving workability, reducing bleeding, and increasing strength and durability. However, excessive nano-silica may lead to high viscosity and delay in setting time. On the other hand, steel fiber reinforced concrete (SFRC) enhances toughness, ductility, and energy absorption capacity, making it suitable for applications involving high strength and dynamic loads. Nevertheless, SFRC also suffers from issues like difficulties in mixing, increased viscosity when fresh, and potential long-term corrosion problems in aggressive environments. Therefore, an optimal approach is needed to incorporate nano-silica into SFRC to maximize the advantages while overcoming the limitations of both materials (Supplementary Fig. 1).

This literature review will provide a comprehensive overview of the current knowledge on concrete properties and critically evaluate the research in this area^6^. We will discuss the factors influencing concrete properties and the methods used to test and evaluate concrete properties. The standards and codes ensure concrete structures’ quality and performance^7^. The field of concrete properties faces several challenges, including variability of concrete mixture, environmental conditions, and lack of standardized testing methods. Variability in the composition of a concrete mixture can result in significant differences in its properties. At the same time, environmental factors such as temperature, humidity, and exposure to chemicals and pollutants can affect the durability and performance of concrete^8^. In addition, the lack of standardization in testing methods can lead to inconsistencies and inaccuracies in evaluating concrete properties, hindering the development of new and improved concrete materials^9^. These challenges highlight the need for ongoing research and development in concrete properties to ensure concrete structures’ quality, performance, and safety^10^. In recent years, there have been several improvements in the field of concrete properties. One area of focus has been the development of new and improved concrete mixtures that use alternative binders, such as fly ash, slag, and silica fume, to replace or supplement Portland cement. These alternative binders can improve the properties of concrete, such as strength, durability, and workability while reducing the carbon footprint of concrete production^11^.

Another area of improvement has been using nanomaterials and other advanced additives in concrete, which can enhance its properties and improve its performance under various loading and exposure conditions^12^. For example, the addition of nano-silica has been shown to improve the strength and durability of concrete. At the same time, using microencapsulated phase change materials (P.C.M.s) can regulate the temperature of concrete structures and reduce energy consumption^13^. Finally, there has also been progress in using advanced technologies, such as sensors and monitoring systems, to optimize the curing and maintenance of concrete structures. These technologies can provide real-time data on the properties of concrete, allowing for more effective and efficient management of concrete structures over their lifespan^14^. These developments in the field of concrete properties demonstrate the ongoing efforts to improve the quality, performance, and sustainability of concrete structures. Concrete has several advantages that make it a widely used construction material. One of its key advantages is its strength, which allows concrete to resist forces and load, providing stability and safety to structures. Concrete is also known for its durability. It can withstand environmental factors, such as weathering, chemical attacks, and physical impacts, and maintain its strength and function over time. Some studied the effects of adding granite polishing waste as paste and aggregate replacements on the packing densities and performance of concrete mixes^15^. They found optimal replacement levels for maximizing packing density and strength while reducing cement content. Researchers investigated ternary blending of cement with metakaolin and silica fume to improve binder paste’s packing density and properties^16^. Adding metakaolin and silica fume increased packing density, released excess water, and improved fresh and hardened properties. The roles of particle packing and water coating thickness on the carbonation and strength of γ-dicalcium silicate-based materials were examined^17^. They identified a critical carbonation depth and optimal water coating thickness for maximum strength and CO_2_ uptake. Some studied the combined use of γ-dicalcium silicate and slag for improved carbonation resistance of blended materials. Adding γ-dicalcium silicate to slag blends enhanced CO_2_ sequestration, pore refinement, and surface hardening, with an optimal combination for minimizing carbonation depth^18^. Others proposed a volume-based approach to concrete mixture design for improved accuracy and consistency^19^. They discussed measuring packing density and controlling water/slurry film thickness as a basis for linking fresh properties to hardened performance. Some quantified the effect of varying cementitious paste volume at a constant water–cement ratio on the properties of concrete^20^. Reducing paste volume increased density and strength but decreased workability, which could be compensated by adding superplasticizers or fillers. Researchers developed infilled cementitious composites featuring high sustainability and performance^21^. He optimized fiber-aggregate skeleton packing and ultra-high performance concrete infill to reduce cost/CO_2_ while enhancing strength. Others applied volume-based design to develop ultra-low cement concrete mixtures^22^. Concurrent paste replacement and sand proportioning methods allowed for minimizing cement while maximizing performance. Optimal paste volume and sand content were identified. Researchers revealed the roles of packing density and slurry film thickness in the synergistic effects of adding metakaolin and silica fume^23^. Increased packing density and proper slurry film thickness explained synergistic flowability, strength, and durability improvements. Others studied the effect of rigid steel fibers on aggregate packing density. Fibers caused proportional packing density reduction dependent on volume and aspect ratio^24^. Others studied the effect of rigid steel fibers on aggregate packing density. Fibers caused proportional packing density reduction dependent on volume and aspect ratio. Larger fibers and smaller aggregates showed greater packing disruption. Using finite element analysis, Ref.^25^ developed an improved pullout test method for rebar in plain/fiber concrete. Inserting a rubber pad and PTFE film reduced testing errors from uneven contact pressure and friction. Others proposed a new bond model for rebar in steel fiber-reinforced concrete^26^. They modified the Model Code 2010 to incorporate fiber effects and finite initial stiffness for realistic bond-slip analysis. Researchers studied the co-addition of metakaolin and silica fume in mortar. Metakaolin improved workability, and silica fume reduced it, but their combination mitigated negative effects while synergistically enhancing strength^27^. Others used wet packing method to measure packing densities of cement-pulverized fuel ash-micro silica ternary systems^28^. Limitations of the Andreasen and Andersen model were revealed through comparison. Researchers recycled waste glass powder by substituting paste volume in UHPFRC, maintaining composition^29^. Optimal glass powder enhanced workability, strength, and microstructure while permitting cement content reduction. Others optimized recycled aggregate proportioning in concrete blocks to increase packing density and match natural aggregate performance without increasing cement^30^. Packing-based prediction equations were derived. Some developed a new direct tension test method for fiber-reinforced concrete using dumbbell specimens. Analysis showed reduced stress concentrations. Fiber effects on first-cracking and post-cracking behavior were quantified. Some extended the water film thickness concept to seawater cement paste^31^. At similar film thickness and superplasticizer, seawater decreased workability but slightly increased strength compared to freshwater paste. Reference^32^ modeled the combined effects of water film thickness and fiber factor on the workability of polypropylene fiber reinforced mortar. This revealed the dominant roles of water film thickness and fiber factor. Reference^33^ studied the effects of different fiber materials and volumes on fiber-reinforced concrete's strength and fracture properties.

This literature review comprehensively examines concrete properties and their associated evaluation techniques. Many factors influence concrete characteristics, including composition, curing conditions, environmental variables, and loading and exposure conditions^34^. One of the central themes of this review is the challenges associated with concrete properties, such as the inherent variability in concrete mixtures, the impact of environmental conditions, and the absence of standardized testing methods^35^. On the flip side, this survey also underscores the significant progress being made in the field of concrete properties. This encompasses the development of novel and enhanced concrete mixtures employing alternative binders, incorporating nanomaterials and advanced additives, and utilizing cutting-edge technologies like sensors and monitoring systems to optimize the curing and maintenance of concrete structures^36^. Concrete stands out as a construction material for its numerous merits, including strength, durability, versatility, affordability, and fire resistance. Nonetheless, further research is imperative to fine-tune concrete properties, ensuring concrete structures’ quality, performance, and safety. The core aim of the study was to scrutinize the impact of adding fibers and nanoparticles on the mechanical attributes of concrete. The investigation assessed compressive and flexural strength, tensile strength, modulus of elasticity, and failure patterns in concrete with varied fiber and nanoparticle dosages. The ultimate objective was to pinpoint the optimal dosage of fibers and nanoparticles that would augment the mechanical properties of concrete, thus contributing to the development of more resilient construction materials. Integrating Nano-Silica in concrete has diverse effects on its fresh and hardened properties, including enhanced workability, reduced bleeding, and improved mechanical characteristics^37^. Nevertheless, it’s worth noting that excessive nano-silica can result in increased viscosity and extended setting time, presenting handling challenges. While bolstering several properties, steel fiber-reinforced concrete (SFRC) has constraints, such as potential non-uniform distribution, increased viscosity, and corrosion concerns in harsh environments. This study delves into the mechanical attributes of concrete mixtures with varied compositions and fiber reinforcements^38^. It’s divided into two phases: Phase 1 evaluates compressive strength, flexural strength, and modulus of elasticity with diverse additives, while Phase 2 explores the effects of increasing fiber dosage on multiple mechanical properties and fracture energy. This research employs standardized testing methods and specimen types. It ultimately compares outcomes across different mixtures, seeking to provide valuable insights into the application of fiber-reinforced concrete in structural engineering.

## Materials and methods

As mentioned, the benefits of incorporating nanoparticles and steel fibers into composite materials are evident, among which the high energy absorption capacity, significant gains in tensile strength and increased ductility, and compressive strength and compactness stand out. However, the choice of the % of addition of fibers to be included in the mixture should be reasonable and follow the mechanical properties of the fibers and matrix. This chapter presents the description and characterization of the constituent materials of MRFAIN, as well as the methodology of their composition. The preparation, curing, and testing of specimens are also described. This study aims to obtain the mechanical characterization of a composition of the high-strength concrete matrix that is considered suitable for self-compacting concretes and for which the following tests were performed: compressive strength test, and tensile strength test (bending). A more complete characterization was made of specimens with the mixture of reference already studied. In addition to the tests already mentioned, the following was the determination of fracture energy modulus of elasticity test. With this set of tests, it is possible to characterize the behavior of this type of concrete that the trials in this phase of the study were carried out for 28 days of age concrete. It should be noted that the complementary tests were performed in the mixtures whose results presented the lowest deviations and more coherent values with the desired performances. For the study of the mixtures, the concrete matrix was considered, consisting of the respective mortar matrix (binders, water, and adjuvants) and fine sand, adding the nanoparticles and fibres to the reference mixtures previously characterized.

Preliminary tests were first conducted on mortar mixtures with different combinations of cement and supplementary cementitious materials (SCMs) including silica fume, metakaolin, and limestone powder (mixtures A–E). Based on workability, compressive strength, and drying shrinkage results, mixture D with 10% metakaolin and 5% silica fume was identified as the optimal SCM blend. Mixture A with 100% cement was also selected as a benchmark for comparison. The hooked-end steel fibers were sourced from multiple suppliers. Since the mechanical properties can vary depending on manufacturing processes, the ranges reported reflect the typical values provided by the suppliers rather than measured data.

To optimize the nano-silica content for particle packing density enhancement, a range of nano-silica replacement levels from 1 to 5% of cement weight were evaluated. The particle size distribution of the cement, silica sand, fibers, and nano-silica were measured by laser diffraction analysis. Packing density modeling was then performed to determine the nano-silica dosage required to fill the voids between the larger particles. The mix proportions were developed based on a modified absolute volume method. For a target coarse aggregate content of 300 L/m^3^, the amounts of all other mix components in the micro-concrete matrix were multiplied by 0.7 to determine their quantities for the concrete-containing aggregates. To ensure uniform dispersion, the nano-silica particles were dispersed in the mixing water using ultrasonication for 15 min before mixing. Proper dispersion of nanoparticles was verified by scanning electron microscopy imaging of the concrete microstructure.

### Binders

Due to cement’s importance in concrete, your choice must follow the desired resistance performance. The cement chosen for this work was the CEM I 52.5 R with a volume mass of 3.12 kg/dm^3^. This grade of cement allows concretes with higher resistances than the more current cement. The additions aim to improve the characteristics and performance of the specified concrete. In this sense, H.D. smoke silicas (volume mass of 2.2 kg/dm^3^), Comital limestone filer (volume mass of 2.7 kg/dm^3^), f-type fly ash (mass volume of 2.3 kg/dm^3^), and Sibelco silica flour (volume mass of 2.6 kg/dm^3^) and Silica flour from Sibelco (volume mass of 2.6 kg/dm^3^). The choice of smoke silicas and fly ash was based on the desired performance increase for concrete. These polyzoan additions increase mechanical strength and better durability, provided that an adequate cure is performed. The use of lime filer and silica flour was due to the objective of considering the increase in the volume of ligating powder without increasing the desired cement act, which has the function of the filer by filling the voids, and the second has a reactivity effect on the cement matrix.

### Aggregates

**Figure 1:**
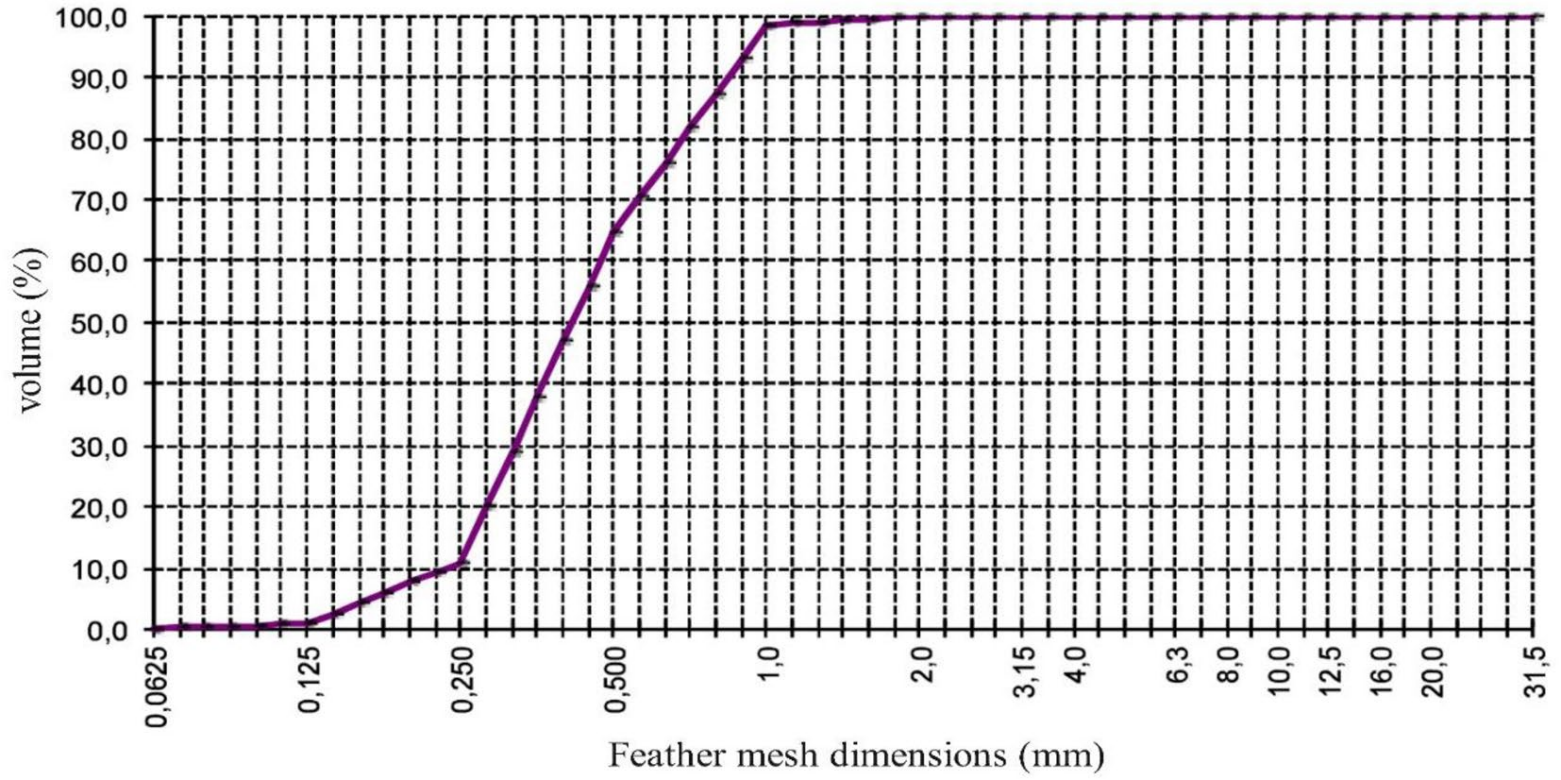
Fine sand granulometric curve 0/1 mm.

The usual normal density aggregates in concrete production are usually of granite or limestone origin. Its classification depends on the granulometry of the material and is divided into fine aggregates in the case of sand and coarse aggregates for gravel. Despite the influence of sand granulometry on the properties of mortars and concrete, particularly in compactness and workability, it was decided to use only fine sand to produce microconcrete. Consequently, of MRFAIN, the normal density aggregates used are usually thin. The studied matrix can constitute the resistant binder fraction and any self-compacting mixture; moreover, it is the fine aggregates that mainly influence the workability and the need for water and additives, the compactness, and the air content, so the behavior can then be extrapolated to concrete with coarse aggregates of the equivalent matrix. In the present work, only one type of aggregate of normal age, fine sand 0/1 mm (volume mass of 2.63 kg/dm^3^), is utilized. Figure 1 shows the siliceous sand with regular granulometry used in this investigation, which allows for adequate workability for the different mixtures.

### Fibers

**Figure 2:**
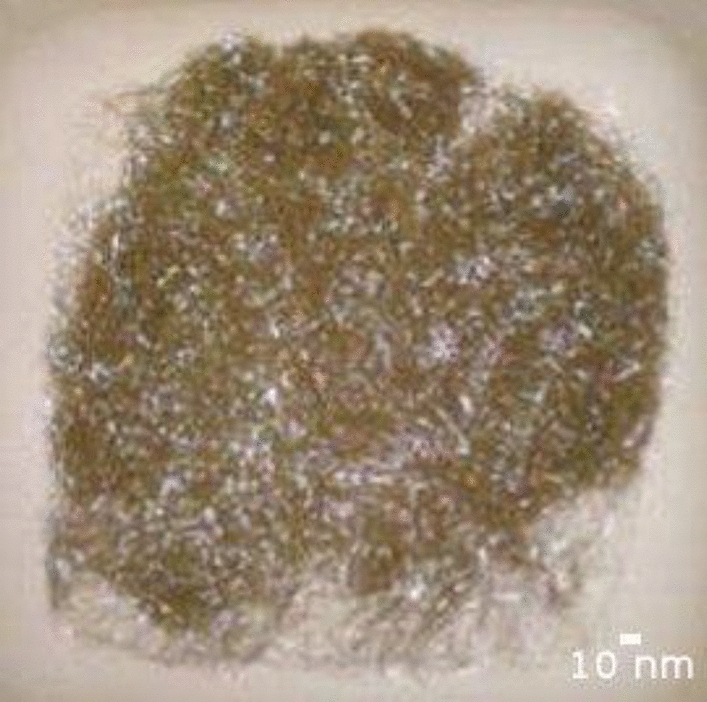
Micro-fibers 0.12/10 mm by Dramix.

Dramix brand steel micro-fibers with a high carbon content and commercial designation 0.12/10 were introduced into the mixtures, representing the nominal dimensions, respectively, the diameter and length of the fiber, in mm. The fibers were chosen to study their influence on the workability of the mixtures and resistance of the specimens, as well as the intended effect of nano-silica on the adhesion between fibers and matrix, as shown in Fig. 2.

### Nanoparticles

In this investigation it was initially intended to study the improvements caused by the incorporation of various nanomaterials in the performance of concrete. However, given the rapid development of the Nano-concrete Project, in which this study was conducted more comprehensively regarding mechanical performance and durability, the influence of only the addition of nano-SiO_2_ was addressed in the present work. The nano-silica (SiO_2_) used was produced based on oxides, with a minimum purity of 99.5%. Its density (kg/dm^3^), obtained in the experimental characterization, was 2.22.

**Figure 3:**
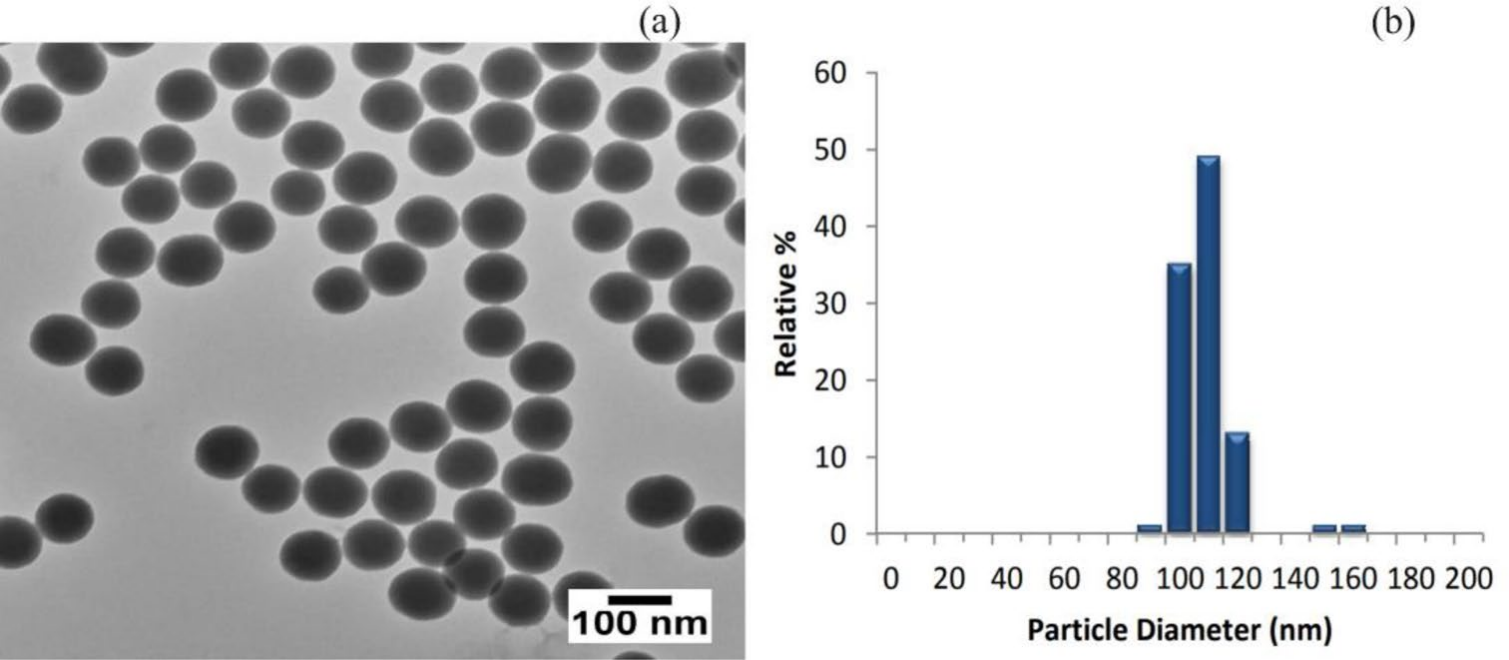
Nanoparticle (**a**) T.E.M., (**b**) particle size distribution.

The diameter of the silica particles was measured using Transmission Electron Microscopy (TEM), is 104 nm, with a first standard deviation of 9 nm as shown in Fig. 3a. The coefficient of variation, at 8.6%, indicates the degree of variation in particle size. A lower percentage suggests greater uniformity, and the particle diameter ranged from 100 to 120 nm, as shown in Fig. 3b.

### Ethical approval

Ethical approval for experiments was obtained from the appropriate institutional review board or equivalent ethics committee, ensuring adherence to ethical guidelines and standards throughout the research.

## Experimental setup and procedure

### Composition methods

The methodology used in the composition of the MRFAIN was based on the philosophy of the composition method. However, since we collaborated only with the binder matrix that contains the fine sand, the granulometric adjustment part of the reference curves is not applied. Thus, the composition study was based on the definition and respective parameters of the binding fraction, as well as the expression of absolute volumes, to control the unitary volume of the mixture. It is also referred to that, considering a fraction of coarse aggregates of around 300 lt/m^3^, the proportions of the micro concrete matrix will be multiplied by 0.7 to obtain the equivalents in concrete with the same matrix and with coarse aggregates. The study was, therefore, based on a dosage of binder (cement and additions) for self-compacting concrete, a value that varies between 550 and 600 kg/m^3^.

Based on what was mentioned above, self-compacting micro concrete mixtures were studied and produced in the laboratory, with a cement dosage of 800 kg/m^3^, adopting a W/L ratio (water/binder) of 0.3; and target air contents of 2.5% to 3% in the mortar matrix.

In each series, five reference mixtures were produced, one with cement (Ci) and the rest with cement and additions: mixture A, with Ci only; mixture C, with Ci and addition of 7% Silica Fume (S.F.); mixture D, with Ci and addition of 20% fly ash (CV); mixture B.C. with Ci and additions of 7% S.F. and 20% Filler (F); mix D.E. with Ci and additions of 7% Silica Flour (F.S.) and 20% CV. As mentioned, the dosage of superplasticizer was adjusted in such a way as to maintain constant, in each series, the workability, measured by the fluidity obtained in the spreading test, and the air content, with the final adjustment of the mixture being conducted in the dosage of sand.

Mixtures A and D were later considered for adding fibers, representing the mixture with cement alone and the mixture with the addition that showed the best performance in preliminary tests. In mixtures A and D, steel microfibers were then added, in volume percentages of 0.5% (5.82), 1% (1.82), 2% (2.82), and 3% (3.82), to analyze the behavior and performance of the mixtures. Based on the results obtained in preliminary tests, it was decided to study only with 1% and 2% of fibers. Then, the study of the mixtures with the addition of the mentioned nanoparticles (nano-silica) was developed, referenced as mixture S, being the addition by replacing the cement, in dosages of 1% and 2%, equally without and with the steel microfibers.

In this experimental procedure, two types of curing were also studied, the normal curing subject to the same type of special curing.

The simplified designation for the mixtures was based on the cement dosage (8) and a certain W/C ratio (2), thus obtaining the base mixture (0.82). The remaining simplified designations of each mixture reflect the variation of the abovementioned parameters, summarized in Table 1.

**Table 1:** Types of binders and fibers.

Binders			Fibers	
	Types	Mixture	%	Mixture
Cement		A	0.5	5
Cement	Filler	B	1.0	1
Cement	Fume silica	C	2.0	2
Cement	Fly ash	D	3.0	3
Cement	Silica flour	E		
Cement	Nanoparticles	S		

### Quantification of aggregates

The quantification of the aggregates is conducted after knowing the absolute volume of the binder paste. The absolute volume of the aggregate mixture is determined based on the absolute volume Eq. (1).

1 $$c+s+a+adj+v+mag=1{\text{ m}}^{3},$$

where c represents cement, s stands for sand, a is used for air, adj denotes admixtures, v symbolizes voids, and mag is defined as aggregates.

2 $$mag={\sum }_{i=1}^{n} a{g}_{i}=1-\left(c+s+a+adj+v\right),$$

where, mag is defined as the absolute volume of aggregates, agi is explained as each aggregate used, n has been defined as the number of aggregates. The absolute volume of aggregates, mag, corresponds to the determination of the sum of aggregates to be used, according to Eq. (2). The absolute volume, mag, refers, in this case, only to the only aggregate used, the fine sand^39^.

### Prediction of mechanical resistance

The prediction of the mechanical resistance of the MRFAIN matrix was determined through the Feret expression as a function of age, considering a correction of the matrix resistance of the binder paste. The calculation of the predicted mechanical resistance consists of estimating the resistance of the binding paste, fc,j, through the Feret expression given in Eq. (3), where K is the Feret coefficient at age j, which depends on the type of cement and the type of dosage is the compactness of the binding paste.

3 $${f}_{c,j}={K}_{1,j}\times {\gamma }^{2},$$

where f_(c,j): Predicted mechanical resistance of the binding paste at age j. K_(1,j): Feret coefficient at age j, which depends on the cement type and the binding paste's compactness. γ2 = degree of compactness

### Mixture composition

The choice of characterized mixtures during phase 1, was based on the study of a reference MRFAIN, with 800 kg/m^3^ of cement, without the addition of fibers and nanoparticles, with a predicted density of 2273 kg/m^3^, assigning it the designation of A0.82. That said, different parameters were varied: types of additions, volumetric dosages of fibers, and volumetric dosages of nanoparticles. In this way, the designations were obtained according to the variations introduced.

Table 2 presents the compositions of the reference mixture and each of the mixtures conducted in the final phase. The composition table consists of the weight quantities of the various constituents, with the sand and adjuvant dosages being adjusted by introducing fibers into the mixture. In the second phase of the final phase study, several types of specimens were made, with varying fibre dosages and two additions that were believed to be the ones that best maximize the performance for the mechanical properties of the concrete under study^40^.

**Table 2:** Concrete compositions for 1 m^3^ of concrete.

Constituent	Assignment	A0.82	BC0.82	BC1.82	BC2.82
		Mass (kg)			
Cement	C.E.M. 1 52.5R	800	800	800	800
Addition 1	Smoke silica	0	56	56	56
Addition 2	Limestone filer	0	160	160	160
Assistant	Glenium sky 526	8	13	14	14
Water		240	240	240	240
Fine Sand	0/1 guide	1226	991	963	934
Fibers	Dramix 0.12/10	0	0	79	157

Table 3 presents the mechanical properties of steel fibers commonly used in fiber-reinforced concrete. Steel fibers are known for enhancing the mechanical performance of concrete structures. The Table summarizes key properties, including tensile strength, aspect ratio, diameter, and specific gravity of the steel fibers. The data shows that steel fibers exhibit high tensile strength, ranging from 1000 to 2500 MPa, contributing to the improved crack resistance and ductility of concrete. The aspect ratio of the fibers varies between 30 and 100, indicating their elongated shape that promotes effective load transfer and bonding with the concrete matrix. The fiber diameter typically ranges from 0.25 to 1.00 mm, while the specific gravity falls within the range of 7.8 to 7.9. These mechanical properties make steel fibers favorable for enhancing concrete’s flexural strength, impact resistance, and post-cracking behavior. However, it is important to note that the specific properties of steel fibers can vary depending on the manufacturer and the specific product used. Therefore, selecting the appropriate type and dosage of steel fibers is crucial based on the desired concrete performance and project requirements^41^. The results of the characterization of nano-silica revealed its chemical composition and physical properties. Chemical analysis showed that nano-silica consists mainly of silicon dioxide (SiO_2_) with a high purity of over 99%. Trace amounts of Aluminium, iron, and calcium impurities were also detected. The physical properties of Nano-Silica included its significantly reduced particle size, ranging from a few nanometres to tens of nanometres. It exhibited a high specific surface area exceeding 100 m^2^/g, attributed to its small particle size and high surface activity. The morphology of Nano-Silica varied, with particles displaying spherical, rod-like, or amorphous structures. The density was approximately 2.2 g/cm^3^, similar to conventional silica fume. Additionally, Nano-Silica particles may possess a surface charge, influencing their dispersion and interaction with other particles in a liquid or solid matrix. These characterization results provide valuable insights into the chemical and physical nature of Nano-Silica, enabling its effective utilization in various applications.

**Table 3:** Mechanical properties of steel fibers used in fiber-reinforced concrete.

Mechanical property	Typical value
Tensile strength	1000–1500 MPa
Modulus of elasticity	200–210 GPa
Aspect ratio	40:1–80:1
Diameter	0.2–1.0 mm
Specific gravity	7.8–7.9
Melting point	1450–1535 °C
Density	7850 kg/m^3^

## Methodology

**Figure 4:**
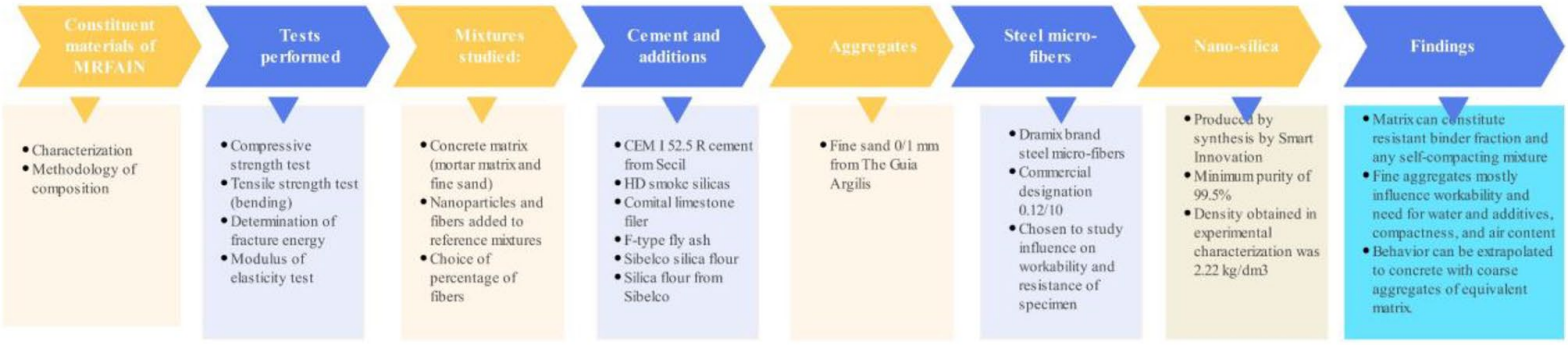
Flowchart to obtain the mechanical characterization of a high-strength concrete matrix suitable for self-compacting concrete.

The experimental plan followed in this study aimed to obtain the mechanical characterization of a composition of high-strength concrete matrix suitable for self-compacting concrete. The addition of nanoparticles and steel fibers into composite materials has shown evident benefits, including high energy absorption capacity, significant gains in tensile strength, increased ductility, compressive strength, and compactness. The choice of the % of fibers to be included in the mixture was informed and followed the mechanical properties of the fibers and matrix. The constituent materials of MRFAIN were described and characterized, and the methodology of their composition was presented. The preparation, curing, and testing of specimens were also represented. The tests performed included compressive strength test, tensile strength test (bending), determination of fracture energy, and modulus of elasticity test. The trials were carried out for 28 days of concrete age, and the methodology is shown in Fig. 4.

For the study of the mixtures, the concrete matrix was considered, consisting of the respective mortar matrix (binders, water, and adjuvants) and fine sand, adding nanoparticles and fibers to the reference mixtures previously characterized. The cement chosen for this work was the CEM I 52.5 R, with a volume mass of 3.12 kg/dm^3^. The additions included H.D. smoke silicas, Comital limestone filer, f-type fly ash, Sibelco silica flour, and Silica flour from Sibelco. The choice of smoke silicas and fly ash was based on the desired performance increase for concrete. These polyzoan additions increase mechanical strength and better durability, provided that an adequate cure is performed. The use of lime filer and silica flour was due to the objective of considering the increase in the volume of ligating powder without increasing the desired cement act, which has the function of the filer, by filling the voids, and the second has a reactivity effect on the cement matrix^42^.

Only one type of aggregate of normal age, fine sand 0/1 mm of The Guia Argilis (volume mass of 2.63 kg/dm^3^) was used for the aggregates. The studied matrix can constitute the resistant binder fraction and any self-compacting mixture. Moreover, the fine aggregates mainly influence the workability and the need for water and additives, the compactness, and the air content, so the behavior can then be extrapolated to concrete with coarse aggregates of the equivalent matrix.

Dramix brand steel micro-fibers with a high carbon content and commercial designation 0.12/10 were introduced into the mixtures, representing the nominal dimensions, respectively, the diameter and length of the fiber, in mm. The fibers were chosen to study their influence on the workability of the mixtures and resistance of the specimens, as well as the intended effect of nano-silica on the adhesion between fibers and matrix.

This investigation addressed the influence of only the addition of nano-SiO_2_. The nano-silica (SiO_2_) used was produced by synthesis by Smart Innovation, based on oxides, with a minimum purity of 99.5%. Its density (kg/dm^3^), obtained in the experimental characterization, was 2.22. The methodology used in the composition of MRFAIN was based on the philosophy of the composition method. However, the details of the methods used were not provided.

## Result and discussions

**Figure 5:**
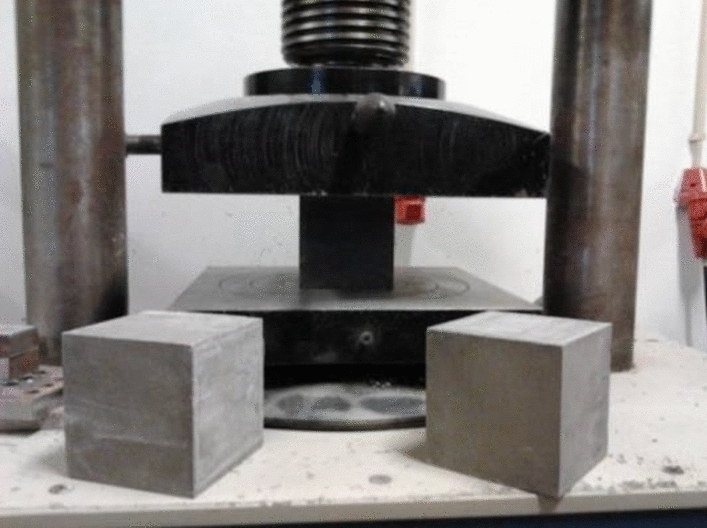
Compression test setup.

In Phase 1 mixtures, the specimens used for this test were the halves resulting from the bending test, with 3 specimens tested at 7 days and 6 specimens at 28 days per mixture. The test is also carried out in the press, using the device with compression plates of 50 × 50 mm^2^, which allows the compression to be applied to each specimen until it fractures. The corresponding compressive strength, f_cm_, is calculated from the specimens’ average breaking strength. In the specimens from Phase 2, the compression test was carried out in a 3000 kN hydraulic press as in Fig. 5, with a constant load application speed equal to 6 kN/s.

**Figure 6:**
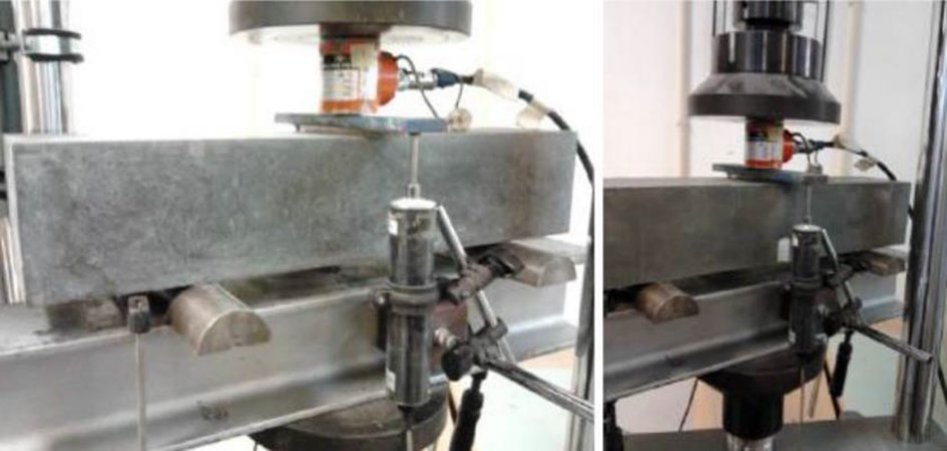
Flexural test and fracture energy setup.

The average compressive strength value, f_cm_, was obtained from the arithmetic mean of the number of specimens. The result obtained is the ratio between the maximum breaking strength and the cross-sectional area of the specimen. The bending test on the prismatic specimens of the Phase 1 mixture was carried out in a universal press, using a 3-point test device, until failure was reached. The flexural strength results, f_cf_, were obtained through the average of three specimens tested at 28 days. For the specimens of Phase 2 mixtures, the bending flexural strength, fcf, was obtained through bending rupture tests and characterized following the NP EN 12390-5 standard. The tests were carried out at 28 days, and the strength was determined for the specimen from the flexion test (cross-section 100 × 100 mm^2^) and the sample from the fracture energy test (cross-section 100 × 66.7 mm^2^). The bending test involves applying a concentrated load at mid-span and along the smallest dimension of the specimen, which is supported by the two central thirds of the span as in Fig. 6.

**Figure 7:**
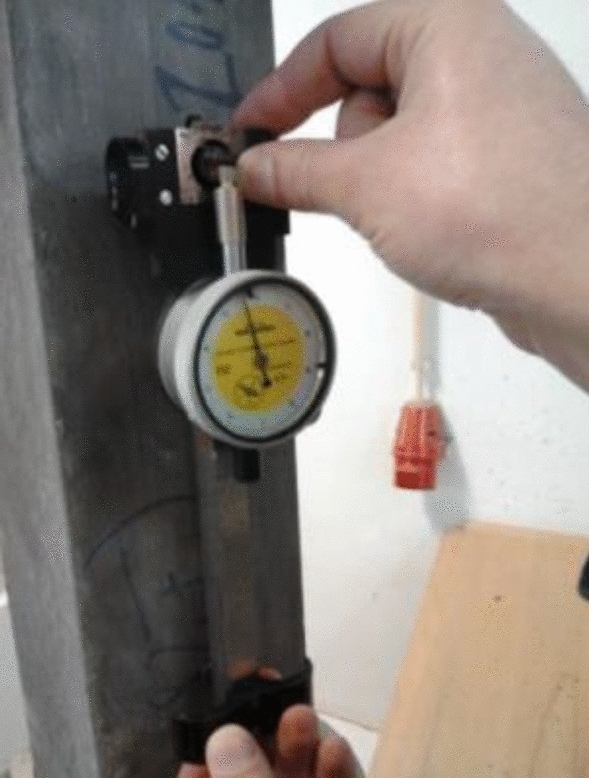
Test to determine the modulus of elasticity in compression.

The static modulus of elasticity was determined according to the specification for prismatic specimens of 100 × 100 × 400 mm^3^. The standard gives that the concrete behaves linearly elastic when subjected to small strains or stresses within a certain range^43^. This assumption is because the elastic modulus of concrete is relatively constant within this range and can be used to accurately predict the deformation and behavior of the material under various loading conditions. The test is based on applying cyclic load on the specimen, between the lower and upper limits, to measure the deformation the sample reaches in each loading and unloading cycle. The loading and unloading speed of the specimens was 0.5 MPa/s. The deformation readings were performed between the two reading points, duly glued to each of the two opposite faces of the sample, as in Fig. 7.

**Figure 8:**
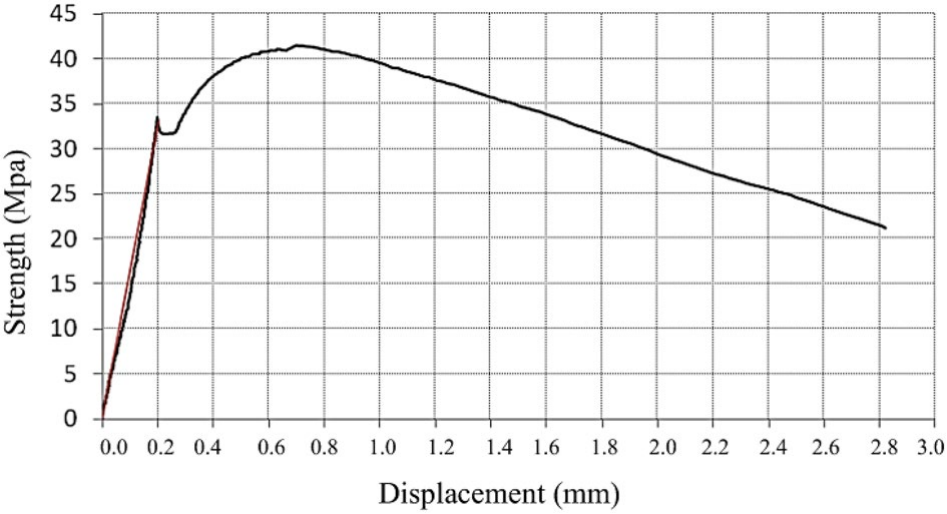
Flexion test chart.

The modulus of elasticity can also be determined in flexion, using the results obtained during the flexion test. During the test, it is possible to decide on the displacement as a function of the applied force, resulting in a graph of the type as in Fig. 8. In the graph, a straight line is fitted to the area with the linear slope. The flexural modulus of elasticity is determined as a function of this slope.

**Figure 9:**
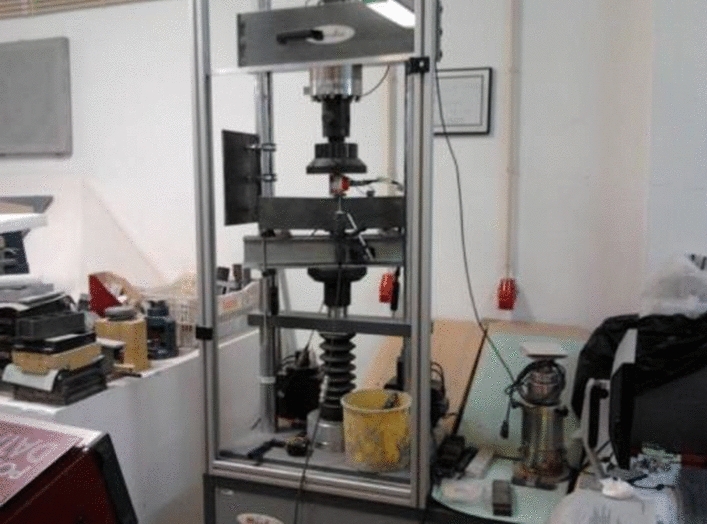
Fracture energy determination test.

The fracture energy test was performed according to the RILEM recommendation in prismatic specimens of 100 × 100 × 50 mm^3^, at 28 days of age. The standard specifies the test conditions and procedures for conducting the freeze–thaw test, including the number of cycles, the temperature range, and the methods for measuring and recording the mass loss of the specimens. The standard also guides the preparation and handling of the specimens and the interpretation and reporting of the test results. This flexural tensile test involves applying a point load at mid-span and measuring the displacements. The specimens were placed on rollers, allowing the beam support points to rotate. The fracture energy is obtained based on the work, W0, performed by the beam during loading. As the failure was intended to occur at mid-span, a notch (100 × 20 × 33 mm^3^) was made in each specimen, per the recommendation above. The test was conducted with deformation control, with the control measure corresponding to the maximum deflection suffered by the beam at mid-span, as in Fig. 9.

As expected, the diameters between 20 and 25 cm were obtained for mixtures with dosages of up to 2% metallic fibers. The expected results regarding the air content were obtained, with slight deviations since the superplasticizer was adjusted to minimize these deviations. The density obtained was initially expected, with close results. As mentioned, the mechanical properties were characterized in the hardened state: compressive and tensile strength, modulus of elasticity, and fracture energy. In Phase 1 mixtures, the specimens used for this test were the halves resulting from the bending test. From the average breaking strength of the specimens, the corresponding compressive strength was obtained, f_cm_, at 28 days, as given in Table 4.

**Table 4:** Compressive strength (Phase 1).

Sample	Strength (Mpa)	Sample	Strength (Mpa)
A0.82N	82.5	DE0.82N	92.3
A5.82N	97.3	DE1.82N	110.9
A1.82N	107.3	DE2.82N	130.5
A2.82N	120.4	DE0.82T	91.9
A3.82N	139.4	DE1.82T	141.5
A0.82T	83.5	DE2.82T	155.1
A1.82T	115.5	BC0.82N	87.2
A2.82T	134.6	BC1.82N	104.6
C0.82N	89.1	BC2.82N	138.5
C0.82T	89.4	BC0.82T	88.3
C1.82T	117.7	BC1.82T	115.7
C2.82T	136.8	BC2.82T	146.2
D0.82N	88.1	AS1 0.82T	88.9
D5.82N	104.2	AS1 1.82T	99.5
D1.82N	114.2	AS2 0.82T	90.3
D2.82N	116.6	AS2 1.82T	107.9
D3.82N	140.7	DES1 0.82T	104.1
D0.82T	84.6	DES1 1.82T	142.4
D1.82T	144.8	DES2 0.82T	101.7
D2.82T	159.0	DES2 1.82T	122.0

For the Phase 2 mixes, after conducting the test on the three specimens, the following results were obtained for compressive strength, f_cm_ at 28 days, as given in Table 5.

**Table 5:** Compressive strength (Phase 2).

Sample	Strength (Mpa)
BC0.82F	107.3
BC1.82F	118.2
BC2.82F	122.5

**Figure 10:**
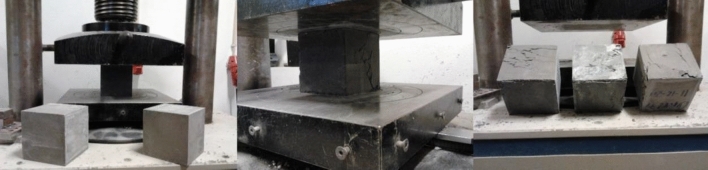
Compression test.

There was an increase in compressive strength in all mixtures with the increase in fiber dosage, which was very noticeable in most situations. In the test specimens, the presence of fibers alters the type of failure, prolonging the test, with a reduced load increase but with a failure that does not generate the detachment of the lateral faces of unconfined concrete. The existing fibers along the fracture lines prevent lateral concrete detachment and show a different failure pattern than the fibreless concrete, as shown in Fig. 10.

As mentioned, the bending test on the prismatic specimens of the Phase 1 mixtures (40 × 40 × 160 mm^3^) was carried out in a universal press, using a 3-point test device, until failure was reached. Flexural strength results, f_cf_ at 28 days, obtained for Phase 1 mixtures are shown in Table 6.

**Table 6:** Tensile strength (Phase 1).

Sample	Strength (Mpa)	Sample	Strength (MPa)
A0.82N	14.1	DE0.82N	7.2
A5.82N	12.5	DE1.82N	24.2
A1.82N	16.1	DE2.82N	33.7
A2.82N	29.3	DE0.82T	15.9
A3.82N	36.0	DE1.82T	20.8
A0.82T	14.0	DE2.82T	36.1
A1.82T	21.0	BC0.82N	9.7
A2.82T	27.8	BC1.82N	17.1
C0.82N	7.8	BC2.82N	30.1
C0.82T	14.2	BC0.82T	14.3
C1.82T	25.6	BC1.82T	22.5
C2.82T	37.9	BC2.82T	32.9
D0.82N	9.5	AS1 0.82T	14.1
D5.82N	12.3	AS1 1.82T	22.7
D1.82N	29.7	AS2 0.82T	14.8
D2.82N	26.5	AS2 1.82T	26.1
D3.82N	34.0	DES1 0.82T	10.0
D0.82T	14.4	DES1 1.82T	22.8
D1.82T	28.7	DES2 0.82T	11.8
D2.82T	37.0	DES2 1.82T	26.0

For Phase 2 mixtures, flexural tensile strength was determined in two tests, a flexural test, and a fracture energy test. The results obtained are shown in Tables 7 and 8.

**Table 7:** Tensile strength (Phase 2 flexion test).

Sample	Strength (MPa)
BC0.82F	9.5
BC1.82F	14.2
BC2.82F	25.4

**Table 8:** Tensile strength (Phase 2 fracture energy test).

Sample	Strength (MPa)
BC0.82F	9.6
BC1.82F	18.9
BC2.82F	18.7

**Figure 11:**
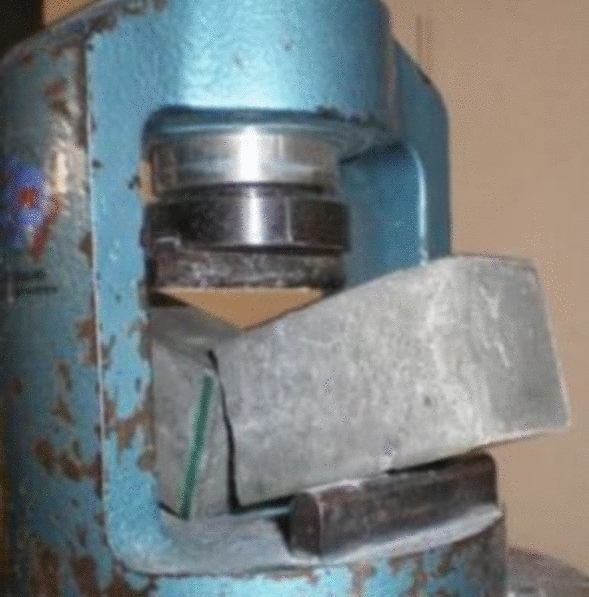
Brittle failure.

**Figure 12:**
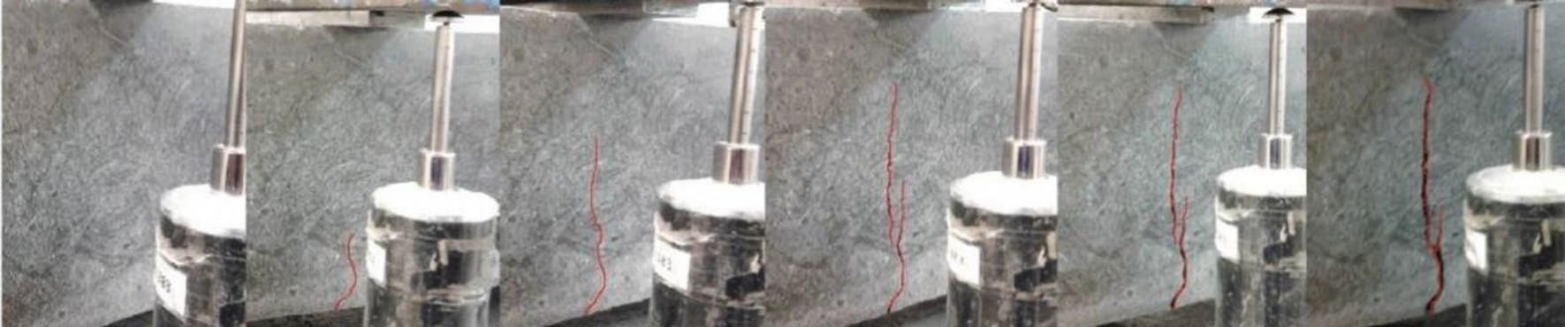
Ductile failure.

Concrete without fibers presents brittle failure, as shown in Fig. 11, concrete with fibers presents a ductile failure, as seen in Fig. 12, reducing the resistance capacity with increased strain.

**Figure 13:**
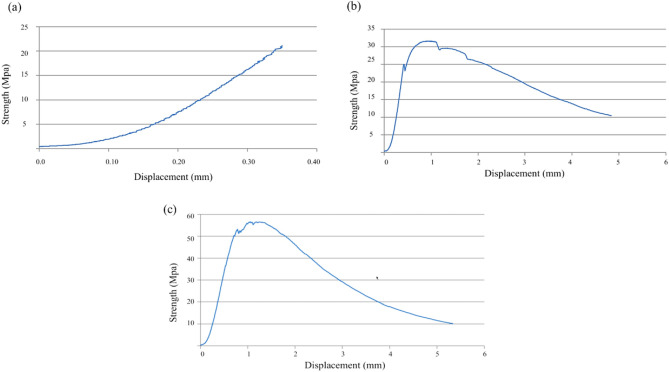
Tensile test (**a**) BC0.82F—0% fiber, (**b**) BC0.82F—1% fiber, (**c**) BC0.82F—2% fiber).

As the % of fibers increases, concrete becomes much more resistant to bending. After reaching the maximum load, concretes with fibers tend to enter a ductile zone, in which the efficiency of resistance and fiber binding is essential to guarantee this ductility, as in Fig. 13.

The introduction of nanoparticles during phase 1, with and without the addition of fibers, proved to be only useful in mixtures with cement alone, even producing an unfavorable effect in mixtures with fly ash and silica flour additions. The modulus of elasticity obtained in compression for the mixtures relating to Phase 2 is shown in Table 9.

**Table 9:** Modulus of elasticity.

Sample	Modulus of elasticity (GPa)
BC0.82F	39.7
BC1.82F	40.4
BC2.82F	42.7

Having conducted the test on the B.C. mixtures, an increase in the modulus of elasticity is verified with the increase in the % of fibers, as expected. The modulus of elasticity in flexion obtained for the mixtures relative to Phase 2, by adjusting the straight line, is shown in Table 10, showing different values compared to the previous ones, being lower in concrete without fibers and higher in concrete with fibers.

**Table 10:** Flexural modulus of elasticity.

Sample	Modulus of elasticity (GPa)
BC0.82F	34.6
BC1.82F	45.2
BC2.82F	45.3

Fracture energy for fiber-reinforced concretes was calculated concerning a specific displacement value, as this test is common for fibreless concretes with brittle failure. A maximum displacement of 9 mm was considered to define the total deformation work, W0^44^. The fracture energy calculation, G.F., was performed. The results obtained for fracture energy are given in Table 11.

**Table 11:** Fracture energy.

Sample	Force (N/mm)
BC0.82F	0.577
BC1.82F	13.071
BC2.82F	12.213

**Figure 14:**
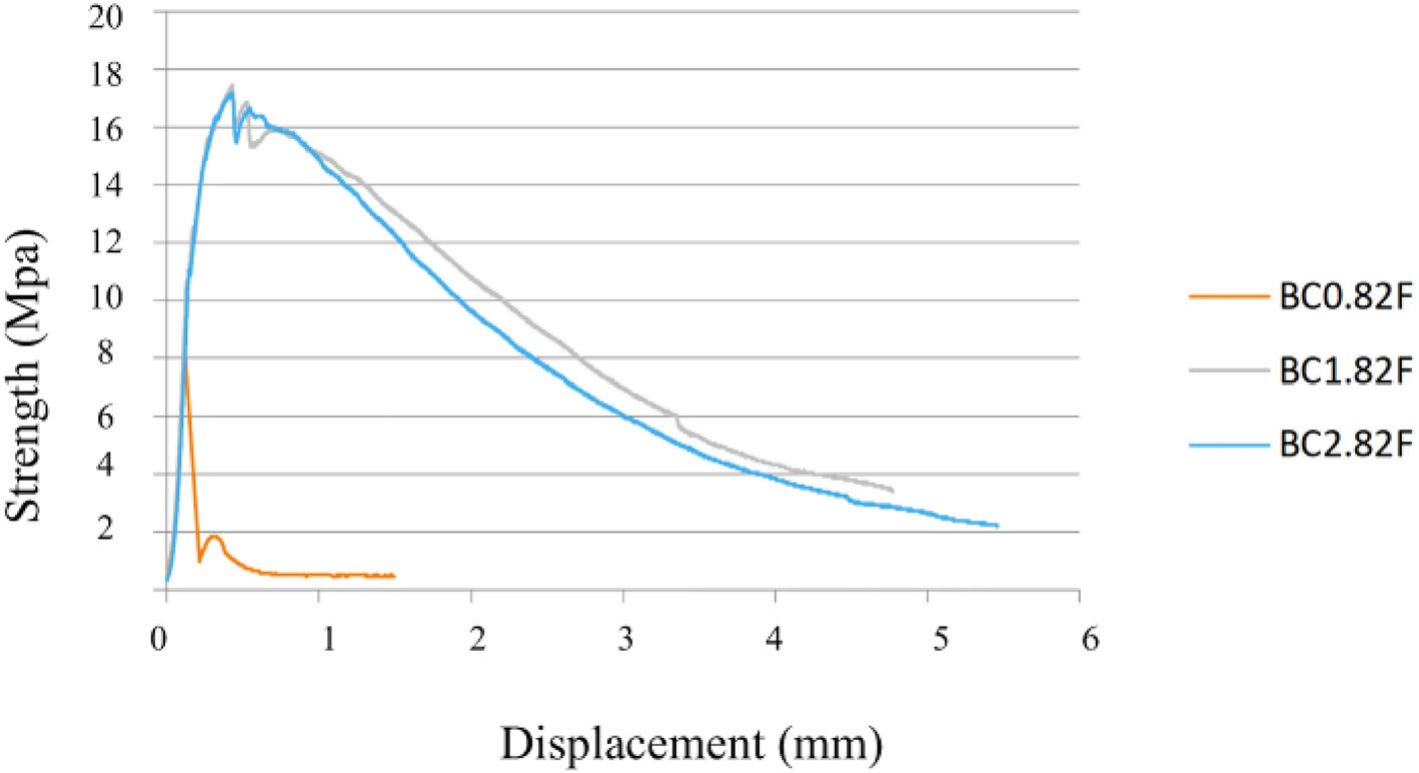
Concrete displacement (fracture energy test).

**Figure 15:**
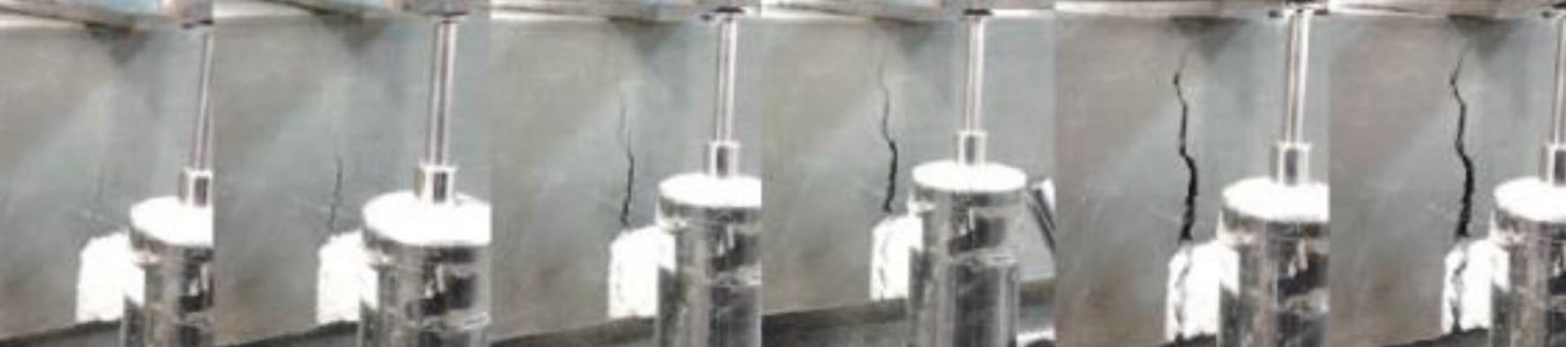
Evolution of the crack in the fracture energy test.

It was found that the greater the energy absorption capacity of the material, the greater the deformation corresponding to the load peak and, consequently, the greater the development of the branch until reaching the maximum load, as in Fig. 14. These results also demonstrate the influence that the dispersion of fibers in the concrete can have, since for a lower % of fibers, a higher Fracture Energy was obtained, indicating the existence of a greater concentration of fibers close to the notch section as in Fig. 15, where the fibers are more efficient, thus leading to a different result than expected. A similar effect was verified in flexural strength^45^. Scanning electron microscopy of the nano-silica concrete indicated a uniform distribution of nano-silica particles throughout the cement paste matrix. No large nano-silica agglomerates were observed, confirming that the ultrasonication technique effectively dispersed the nanoparticles. The uniform nanoparticle dispersion provided nucleation sites throughout the paste matrix, leading to the refined microstructure and reduced porosity observed. Well-dispersed nano-silica enables optimal pozzolanic reactivity and densification of the cement paste gel.

The analysis of the experimental results consisted of (i) evaluating the influence of fibers and nanoparticles on concrete, (ii) study correlations between the properties and parameters studied in the MRFAIN; (iii) comparing experimental results with the codes^46^.

**Figure 16:**
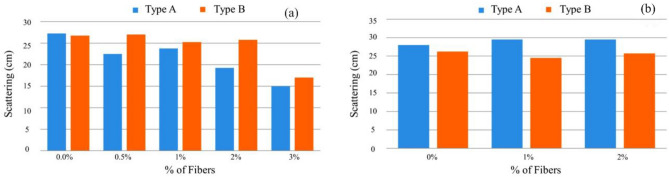
Scattering results for (**a**) Type A and Type B mixtures, (**b**) Type BC and Type DE mixtures.

**Figure 17:**
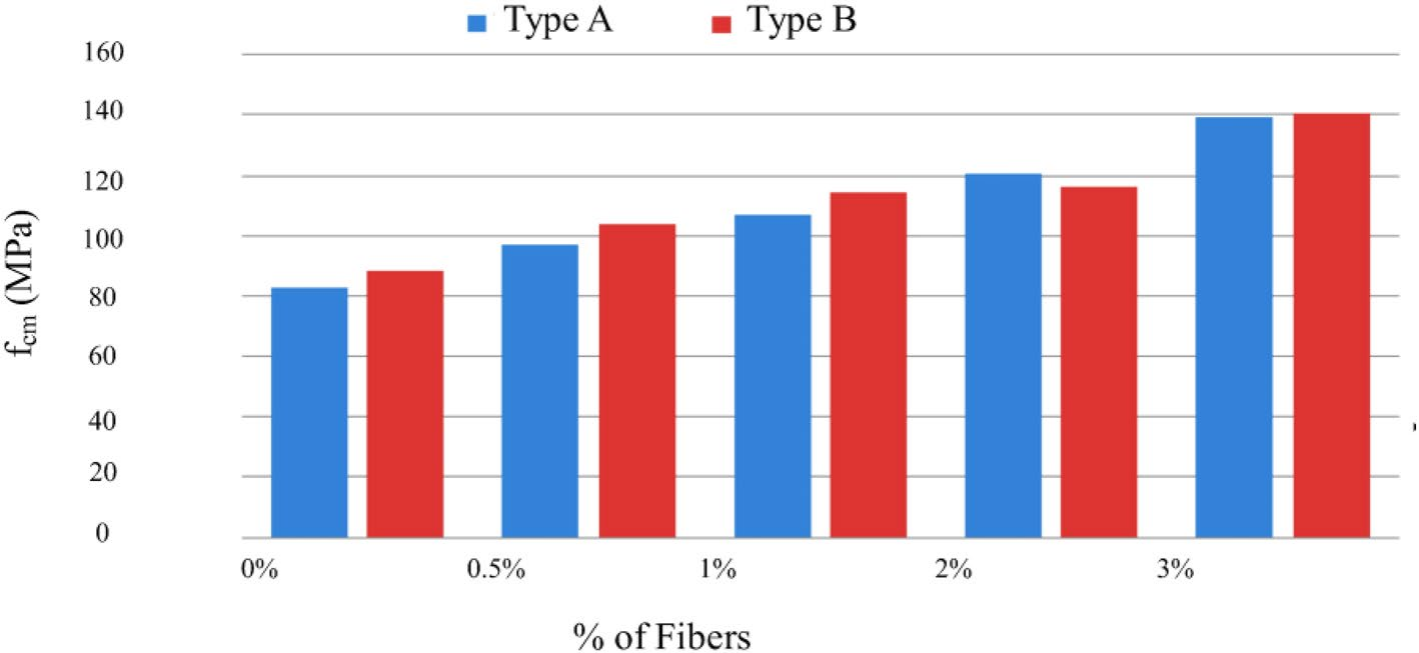
Variation in compressive strength with an increasing % of fibers (Type A and D—normal cure).

The flow test was used to evaluate the fluidity, where the spreading diameter represents a parameter of the fluidity of the mixtures. Figure 16 compares the reference and mixture with just one addition. It is verified that, as the dosage of fibers increases, the spreading diameter only has a marked decrease for a dosage of 3% of metallic fibers. In the same sense, it is observed in Fig. 17 that, for mixtures with two types of addiction, the scattering does not have a notorious variation for mixtures with two types of addiction since the superplasticizer dosage was slightly increased to maintain the air content and spread with the different percentages of fibers. The need to introduce a more significant amount of adjuvant compared to the reference mixture was thus well known to maintain the desired air content and workability^47^. There is a known loss of workability for dosages of 3% of fibers, with the same introduction of fibers affecting fluidity, largely due to their influence on the massing process. The steel fibers exhibited a wide range of tensile strength and elastic modulus values, highlighting the need for tighter specifications on fiber mechanical properties in order to reduce variability in the composite performance.

**Figure 18:**
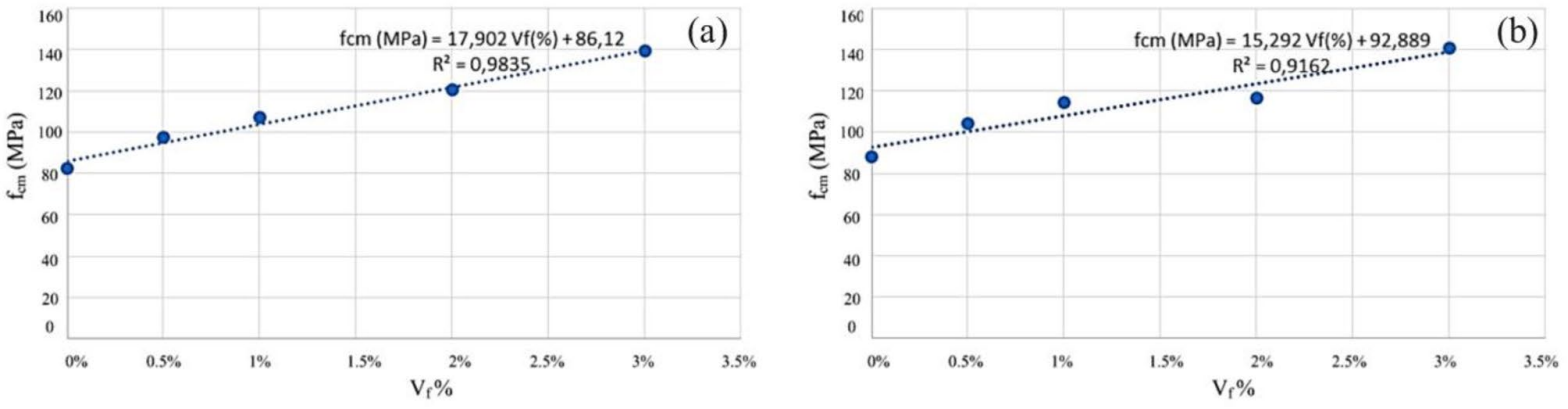
Evolution of f_cm_ with the % of fibers, Vf. (**a**) Type A mixtures, (**b**) Type D mixtures.

In the hardened state, mechanical properties were characterized by compressive and tensile strengths, modulus of elasticity, and fracture energy. The analysis of the results obtained in the MRFAIN assays found that adding fibers and increasing their dosage increased compressive and tensile strength^48^. Adding fibres also increases the modulus of elasticity; concerning the effect of fibers on fracture energy, although the fibers cause a notorious increase, this study is not conclusive of the direct relationship with the dosage of fibers. The test specimens with the presence of fibers changed the type of fracture/breakage; once the maximum load was reached, there was a greater deformation and no total detachment of concrete compared to the concrete without fibers. With the increase in the % of fibers, the rupture mode was increasingly dubious, and a cutting surface was developed accompanied by a deformation^49^. The most notorious differences occurred in concretes with a high % of fibers, which significantly increased their properties in the hardened state and fluidity losses in properties in the fresh state. As expected, the additions and type of cure showed some improvements in the properties of concrete, but the small size of the sampling does not allow the exact of this influence. From the analysis of the results obtained in the MRFAIN trials, it was found that the addition of fibers and the increase in their dosage resulted in a significant increase in compressive strength. In the test specimens, the presence of the fibers altered the type of fracture/breakage, with increased ductility, once the maximum load was reached, there was a greater deformation and without total detachment of concrete, compared to the concrete without fibers. The increase in resistance was more notorious for concrete with 3% fiber (high %)^50^. The following Figures show the evolution of compressive strength, f_cm_, depending on the type of cure and the different rates of fibers and nanoparticles used in the concrete under study. In Fig. 17, there is a good relationship between strength and % of fibers, and compressive strength increases for the studied mixtures by about 18 MPa for a % unit of the volumetric dosage of fibers, thus proving the good correlation shown in Figs. 17 and 18.

**Figure 19:**
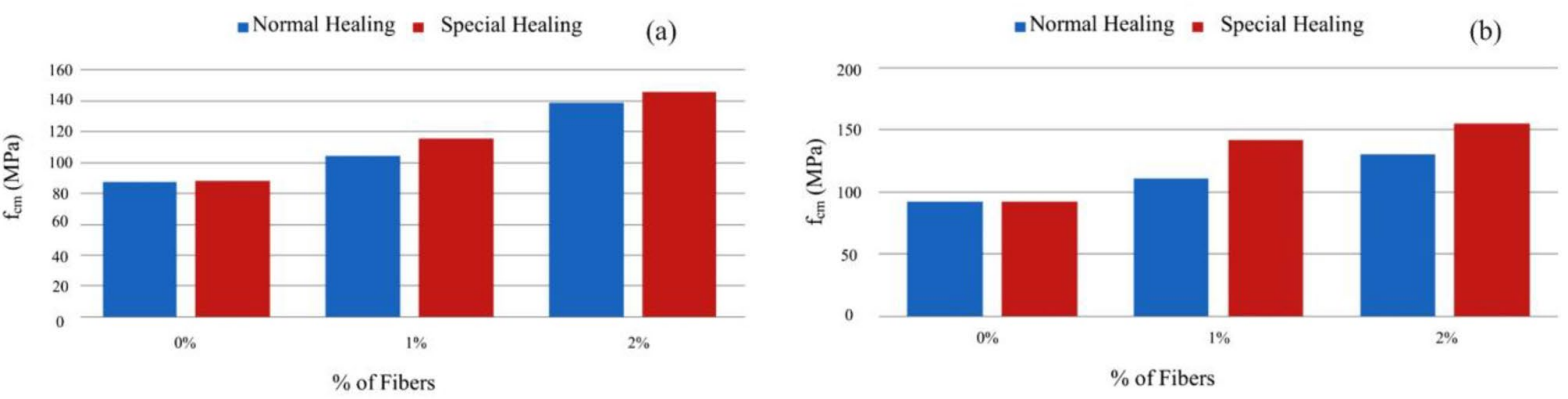
Variation of compressive strength with the variation of the type of cure and with the increase in the % of fibers for a sample of (**a**) Type BC, (**b**) Type E.D.

Figure 19 shows a slight increase in compressive strength with the adoption of a special cure, rather than a normal cure, for the same type of ligament paste, in addition to the influence of increased fiber dosage on compressive strength.

**Figure 20:**
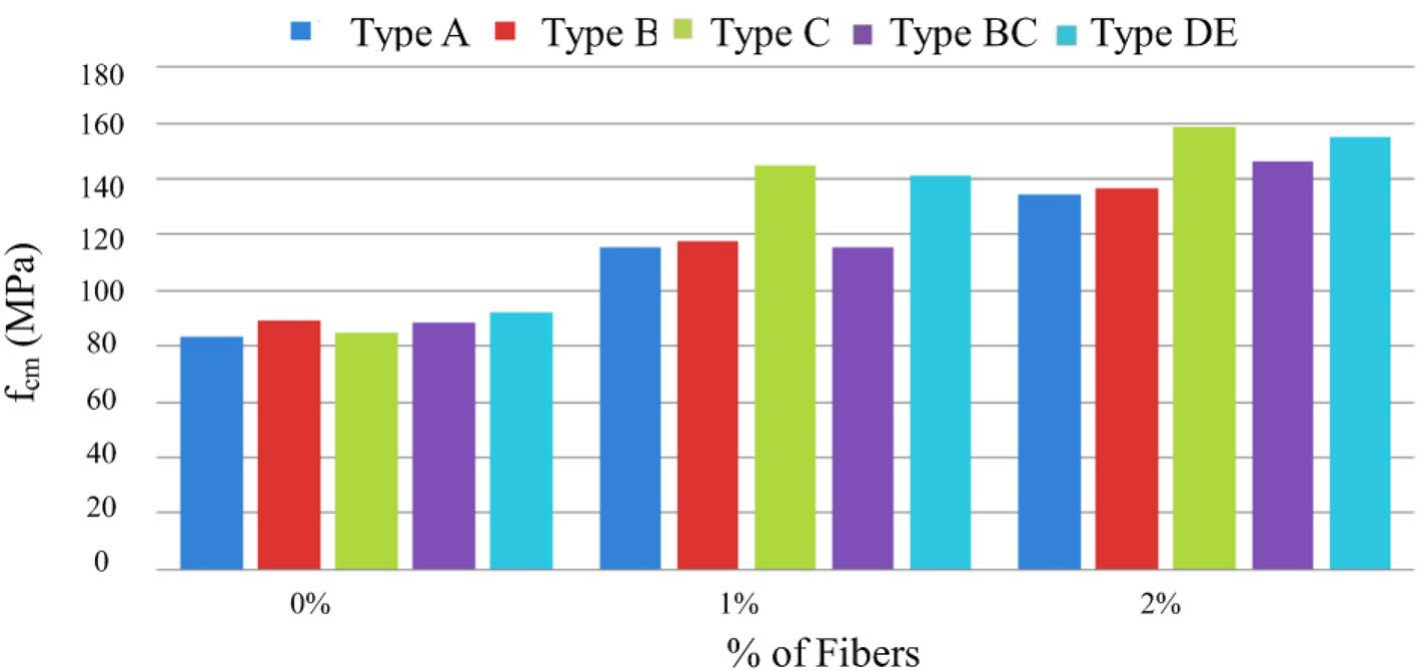
Variation of compressive strength with sample-type variation and fiber % increase (special cure).

Figure 20 shows the trend of increased compressive strength with increased volumetric fiber dosage. The influence of some types of addition can also be observed, which slightly improves compressive strength, namely type C, with the addition of fly ash^51^.

**Figure 21:**
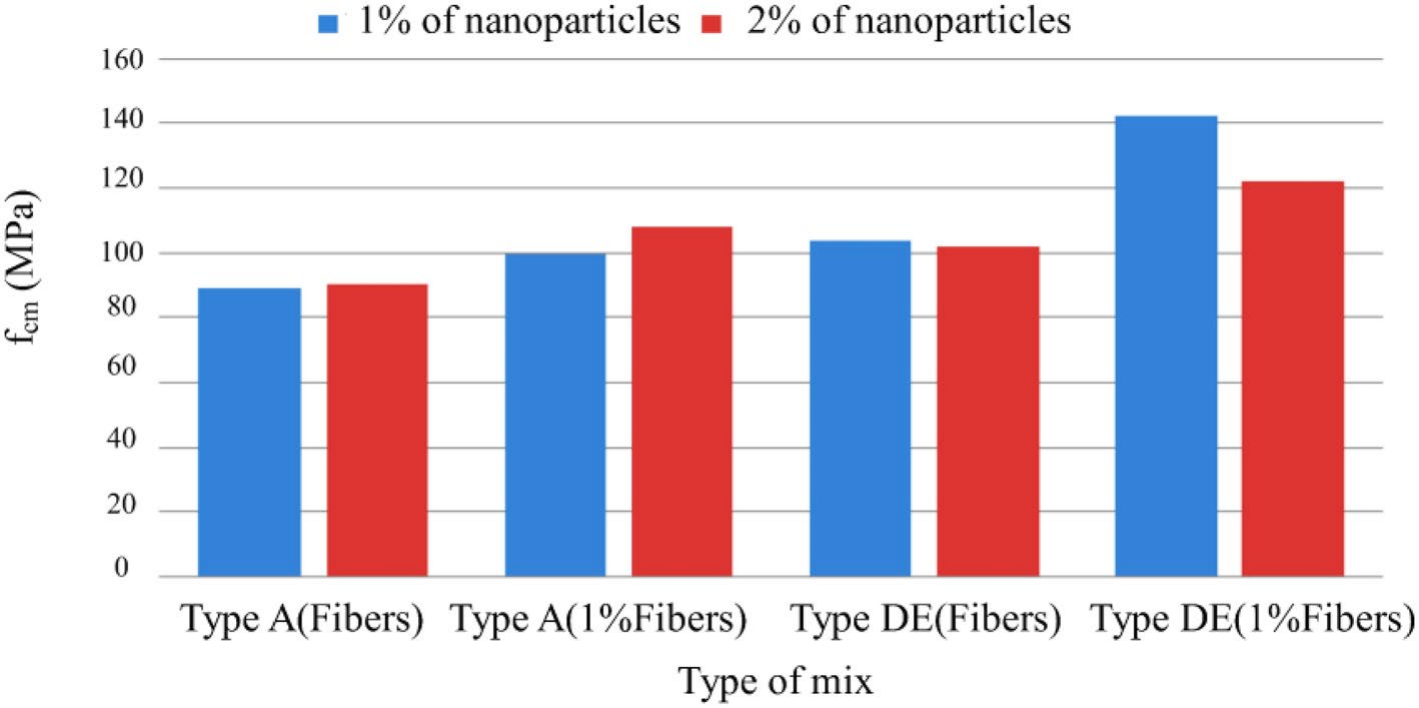
Variation of compressive strength with the variation of the % of nanoparticles and fibers added (Types A and D.E. with special cure).

Figure 21 shows the influence of nanoparticles on compressive strength, which, unlike expected, only has a positive effect for mixtures without additions, being even unfavorable in mixtures with the addition of fly ash and silica flour, most likely because there may be less chemical compatibility between these two additions and the nanoparticles.

**Figure 22:**
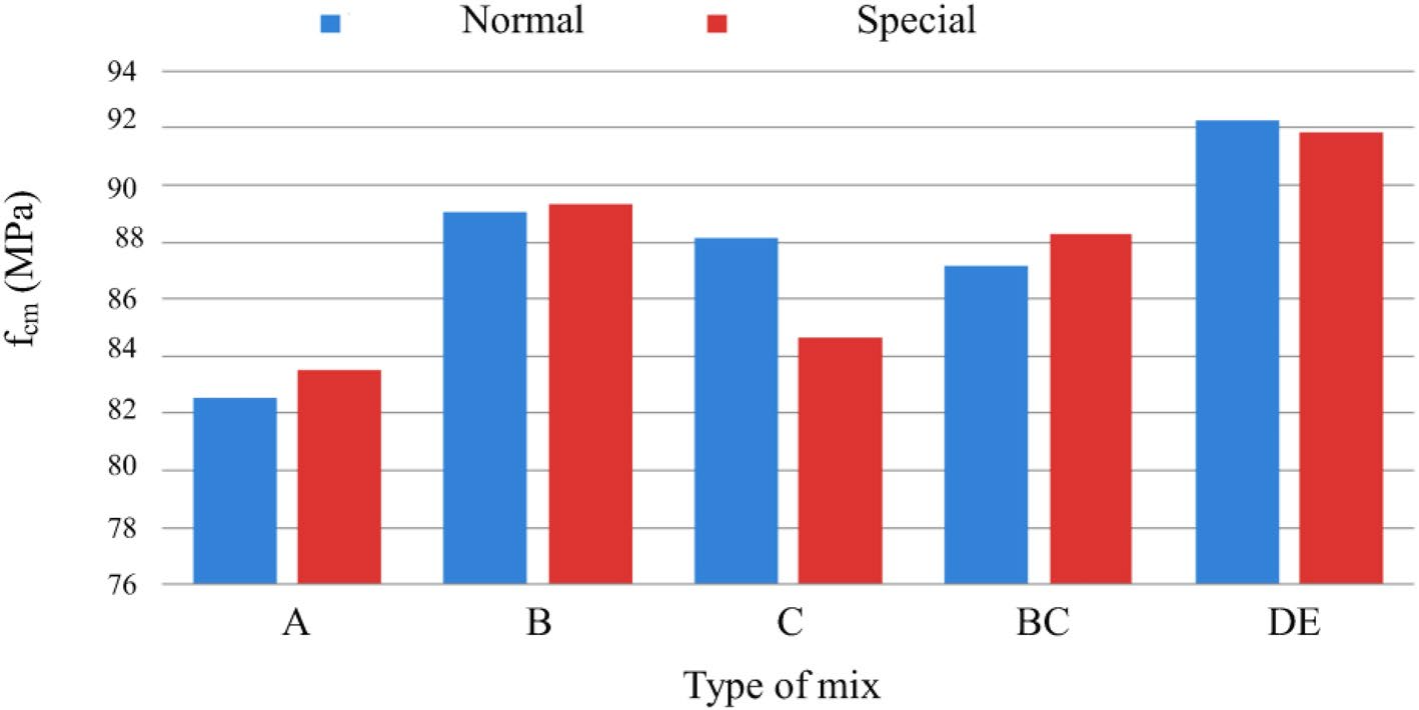
Variation of compressive strength with a variation of cure type and sample type (without fibers).

**Figure 23:**
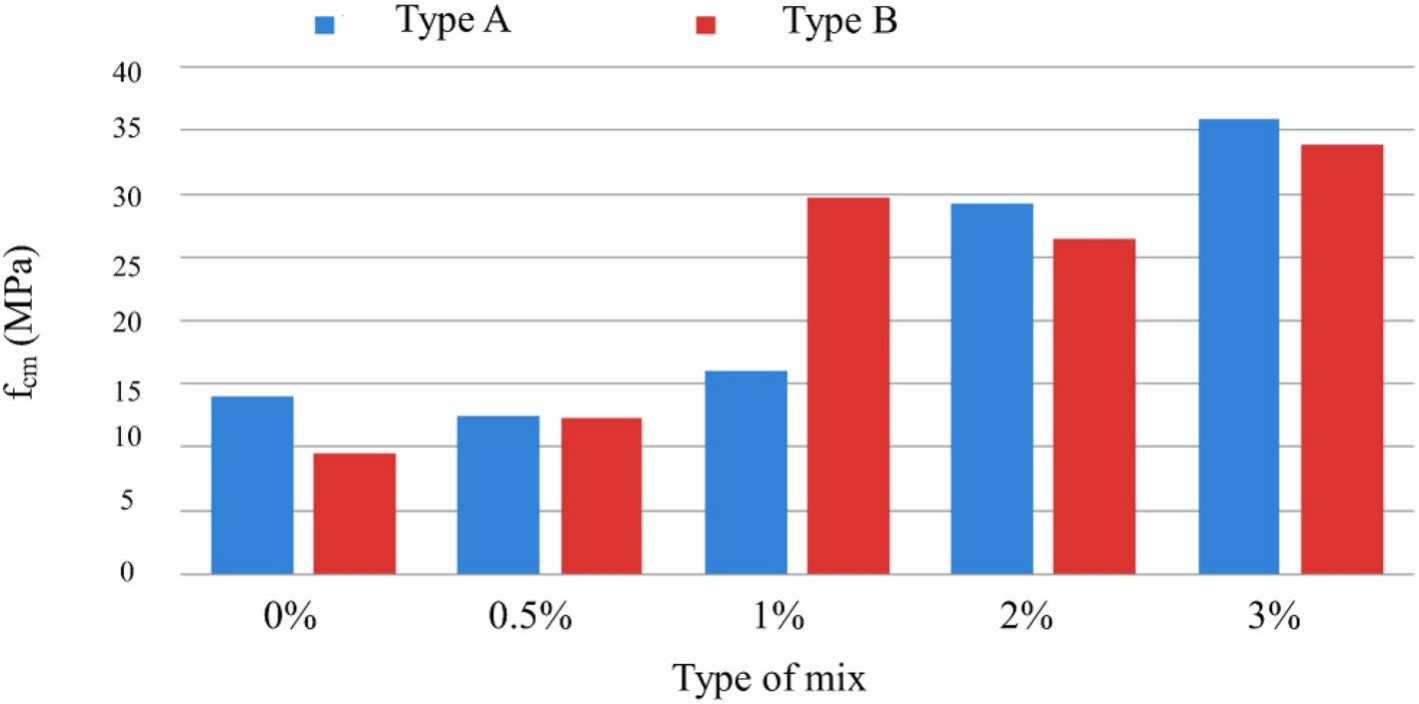
Variation in tensile strength with increasing fiber % (Type A and D—normal cure).

Figure 22 presents results regarding the influence of special cure on the studied mixtures. However, it is expected to increase the compressive strength with the adoption of a special cure, which was notorious for the generality of the mixtures, in the mixture of and especially in D there was a loss of resistance with the special cure^52^. The analysis of the results obtained in the MRFAIN trials found that adding fibers and increasing their dosage increased tensile strength. In the test specimens, the presence of the fibers altered the type of fracture/breakage; once the maximum load was reached, there was a more significant deformation and no total detachment of concrete compared to fibreless concrete^53^. The following Figures show the evolution of tensile strength, f_cf_, depending on the cure type and the different percentages of fibers and nanoparticles used in the concrete under study. Figure 23 shows a good correlation between strength and rate of fibers, and tensile strength tends to increase for the studied mixtures by about 8 MPa for a % unit of fiber volumetric dosage. Mixtures A and D, representing 100% cement and the optimal SCM blend, were selected to add steel fibers to investigate the effects of the SCMs on fiber-reinforced concrete properties. With its high strength and low shrinkage, mixture D was expected to transfer stresses to the dispersed steel fibers better. The pure cement mixture A would allow the influence of the SCMs on fiber contribution to be isolated. Including fibers increased the post-cracking tensile strength and toughness for both mixtures A and D. However, the improvements were more significant for mixture D due to the higher matrix strength and denser microstructure provided by the SCM blend. This demonstrates the synergistic effects between SCMs and steel fibers.

**Figure 24:**
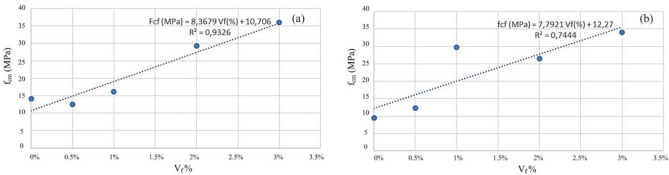
Evolution of fcf with the % of fibers, Vf. (**a**) Type A mixtures, (**b**) Type D mixtures).

Figure 24 shows an increase in tensile strength with adopting a special cure, rather than a normal cure, for the same type of ligating paste, in addition to the influence of increased fiber dosage on tensile strength. The exception was a mixture, where there would have been deviation responsible for the result obtained not expected^54^.

**Figure 25:**
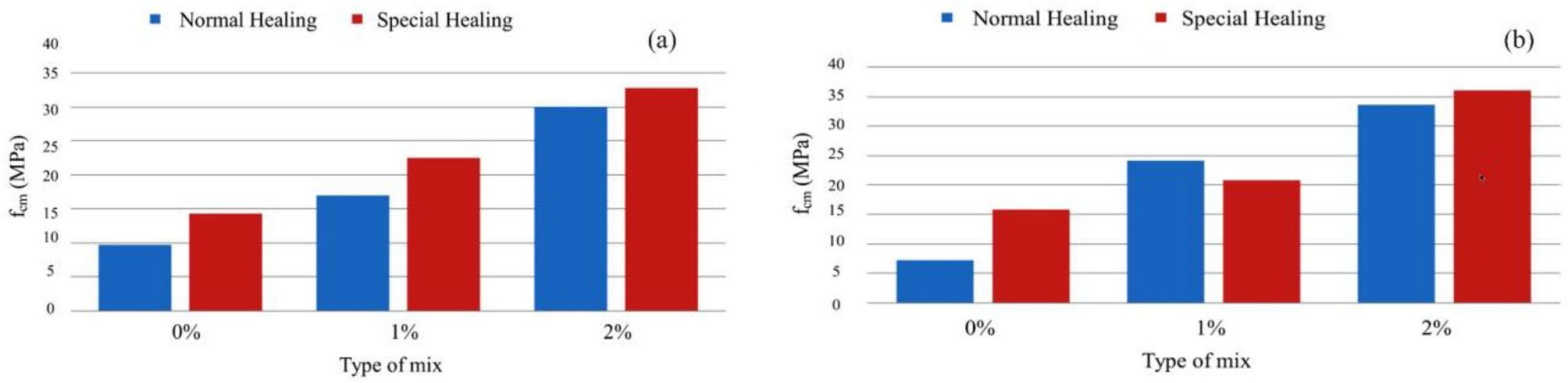
Variation of tensile strength with the variation of the type of cure and with the increase in the % of fibers for a sample of (**a**) Type BC, (**b**) Type E.D.

Figure 25 shows increased tensile strength with increased volumetric fiber dosage. The influence of some types of addition (mixtures B and C with additions of smoke silica and fly ash) can also be observed, which slightly improves tensile strength, but in an inconclusive way, for the sample under study.

**Figure 26:**
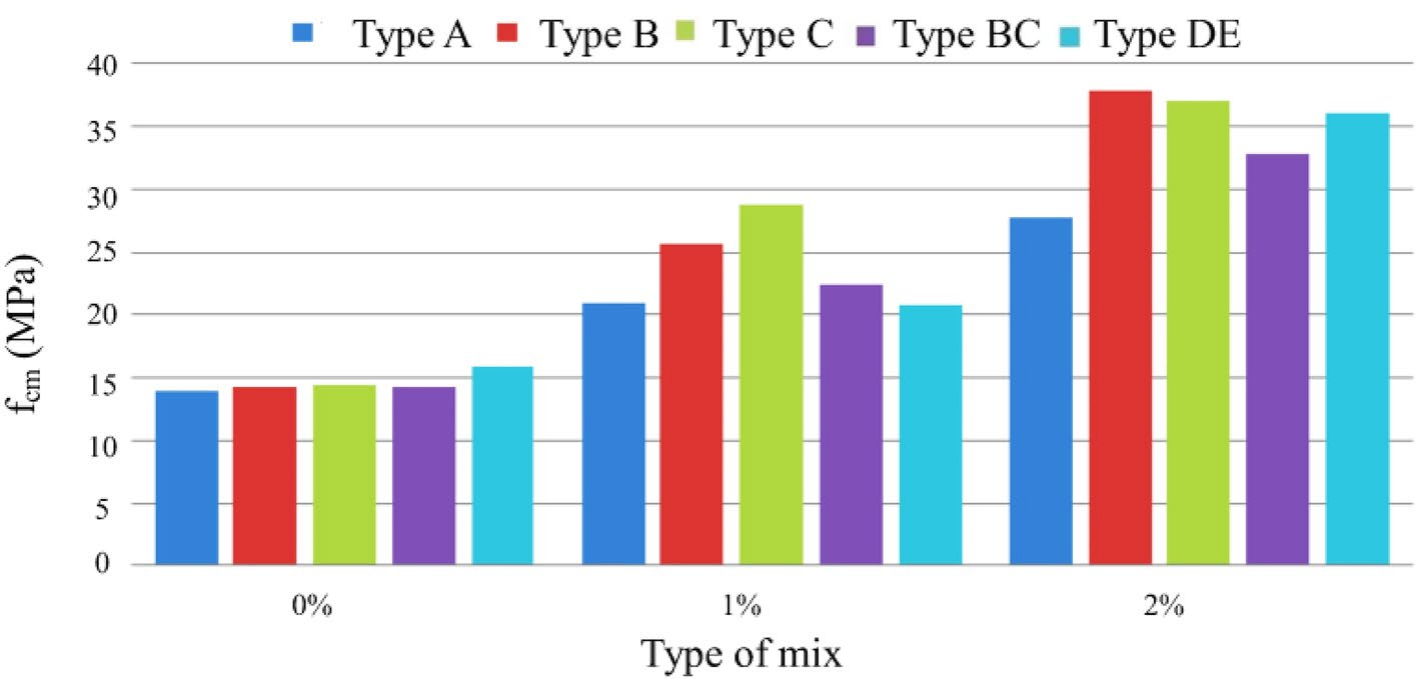
Variation of tensile strength with sample type variation and increase in fiber % (special cure).

**Figure 27:**
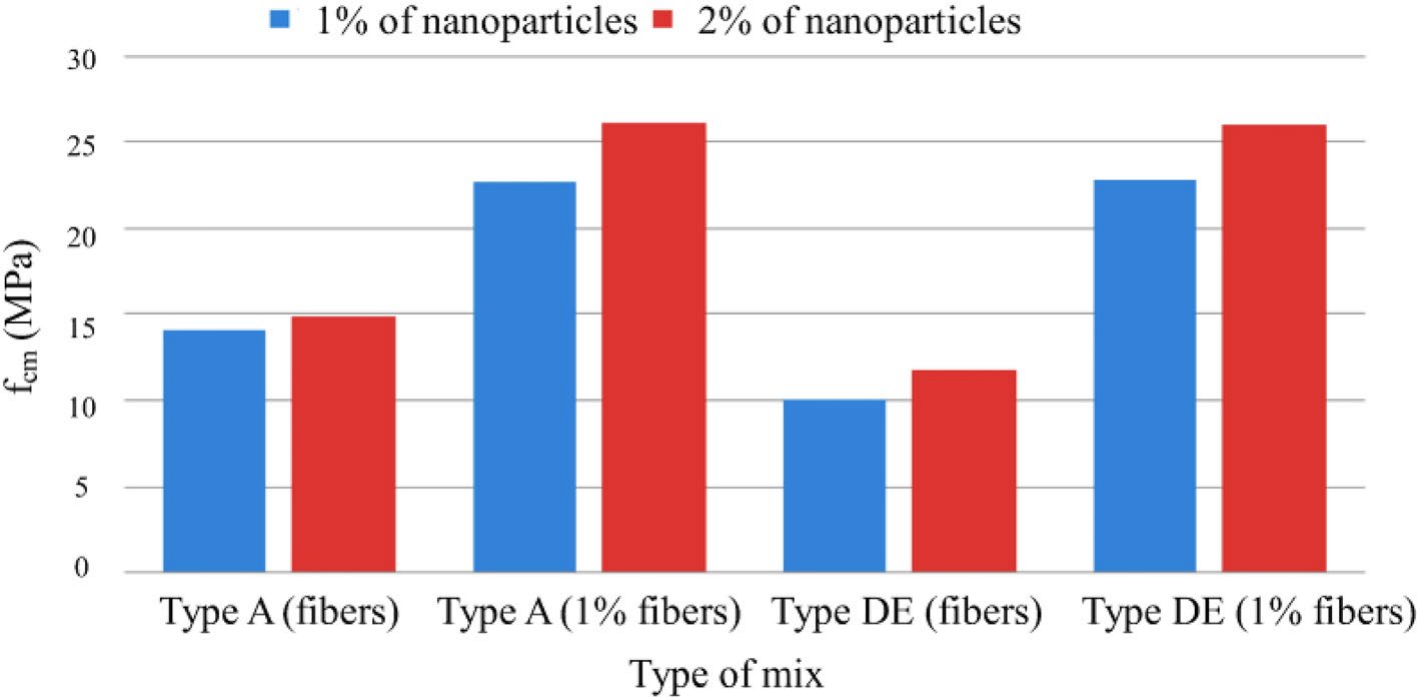
Variation of tensile strength with variation in the % of nanoparticles and fibers added (Types A and D.E. with special cure).

Figure 26 shows the influence of nanoparticles on tensile strength, which, unlike for the study of compressive strength, now has a positive effect for both cases, with the respective increase in tensile strength^55^. Figure 27 shows a notorious increase in tensile strength by applying a special cure in the studied mixtures. Again, it is verified that the additions used in the mixtures in question do not present conclusive results, including an unfavorable result with normal cure in these samples. Using steel fibers from different sources led to a wide range of reported mechanical properties, as seen in Table 3. This fiber variability introduced some uncertainty into the precise composite behavior. For future studies, measuring the tensile strength and modulus directly on the supplied fiber batches could provide more accurate data input for predictive models.

**Figure 28:**
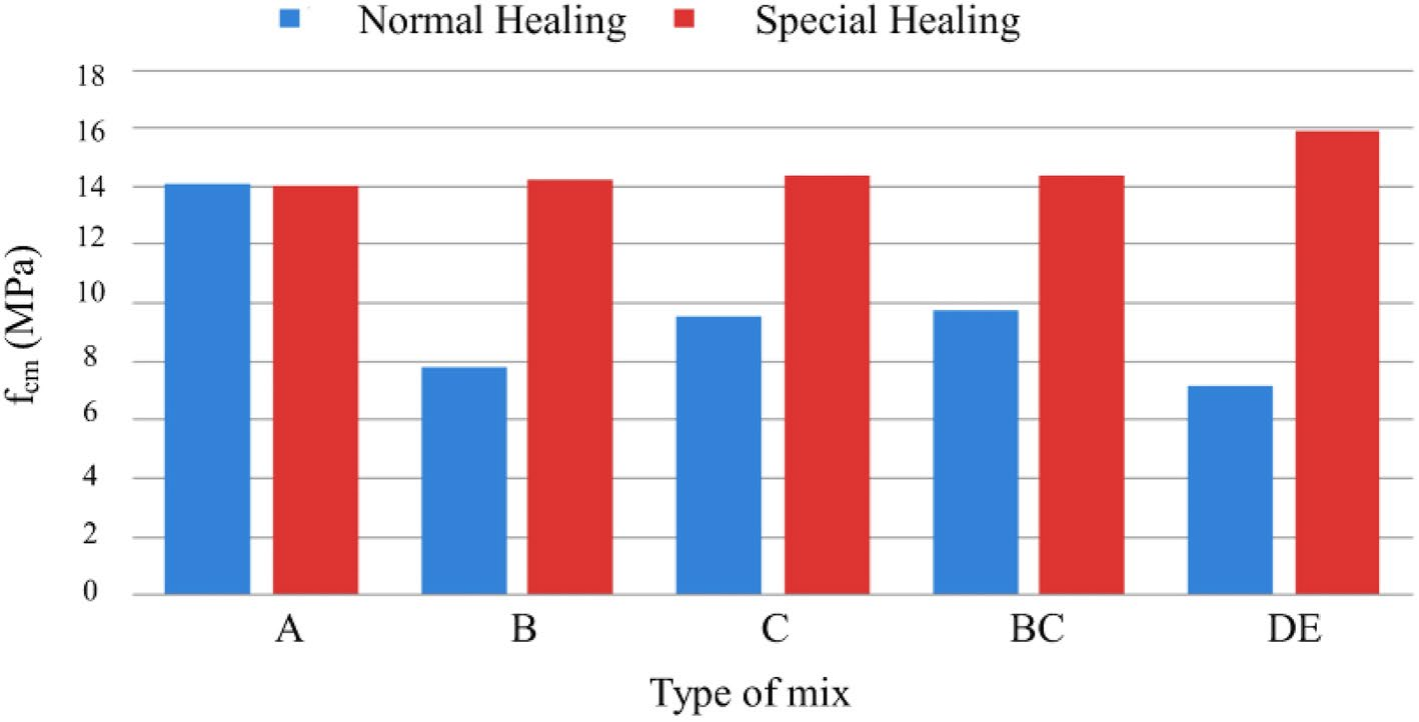
Variation of tensile strength with variation of curing type and sample type (without fibers).

The modulus of *elasticity, E*, was affected by adding fibers in concrete, demonstrating a slight influence of metal fibers on the specimens and their stiffness by the confinement capacity of the matrix, as shown in Fig. 28. The energy absorption capacity increases with the % of fibers. Fracture energy was significantly increased in the concrete scans with fibers compared to fiber-free concrete. Fiber-reinforced concretes require more energy to overcome fibers' reinforcing and mooring mechanisms. The figure shows the test graph in the BC0.82 mixture, which demonstrates the influence of metal fibers on fracture energy^56^.

### Comparison with other studies

From the analysis of the experimental results related to the characterization of the properties of MRFAIN, it was possible to observe the advantage of the incorporation of steel fibers in concretes, both in terms of resistance and ductility; however, unlike some studies already published, the influence of nanoparticles has not proved so notorious and some aspects as conclusive. These results show the already known tendency of the difficulty of generalization of the tests with nanoparticles, given their properties and variation of the reactivity itself in the ligating matrix, by the differences in raw material and the production process of synthesis^57^.

Table 12 compares the mechanical properties of concrete with steel fibers between the current study and previous research. The compressive strength of the concrete mixture containing 2% fibers was determined to be 107.3 MPa in this study, whereas previous researchers reported values ranging from 80 to 100 MPa for the same fiber dosage. The tensile strength measured in this study was 25.4 MPa with 2% fibers, while previous studies reported values between 15 and 20 MPa. The modulus of elasticity in this study was 42.7 GPa with 2% fibers, compared to a range of 30–40 GPa reported by previous researchers. Additionally, the fracture energy measured in this study was 18.7 N/m with 2% fibers, while previous studies reported values between 10 and 15 N/m for the same fiber dosage. These findings suggest that the concrete mixtures in this study exhibited superior mechanical properties compared to previous research, indicating the potential effectiveness of the steel fibers utilized.

**Table 12:** Comparison of mechanical properties of concrete with steel fibers between present work and previous works.

Property	Present work	Previous works
Compressive strength (MPa)	107.3 (2% fibers)	80–100 (2% fibers)
Tensile strength (MPa)	25.4 (2% fibers)	15–20 (2% fibers)
Modulus of elasticity (GPA)	42.7 (2% fibers)	30–40 (2% fibers)
Fracture energy (N/m)	18.7 (2% fibers)	10–15 (2% fibers)

The optimized nano-silica SFRC mixture with 2% nano-silica exhibited reduced bleeding and segregation and a minor viscosity increase compared to the control SFRC. This demonstrated the positive effects of the nano-silica in enhancing particle packing density. The increased packing density refined the pore structure, significantly reducing permeability while maintaining workability. The addition of 2% nano-silica also provided a 12% increase in the 28-day compressive strength. The strength enhancement can be attributed to the nano-silica providing additional sites for forming strength-contributing calcium-silicate-hydrate gel. Thus, the study results aligned with the theory that optimized nano-silica content designed based on particle packing modeling can improve the fresh and hardened properties of SFRC.

## Conclusions

This experimental study provided significant insights into the effects of steel microfibers and nano-silica additions on the mechanical properties of concrete. The key conclusions are:

- Incorporating steel microfibers at higher volume fractions of 1–2% enhanced concrete’s compressive strength, tensile strength, flexural strength, and fracture energy. However, excessive fiber content above 2% led to reduced workability.
- The addition of nano-silica showed variable effects on compressive strength, with an optimal 1% cement replacement dosage. However, nano-silica consistently improved the tensile strength of concrete.
- The combined use of microfibers and nano-silica demonstrated the best mechanical performance, with 2% steel fibers and 1% nano-silica leading to peak improvements in compressive strength (122.5 MPa), tensile strength (25.4 MPa), and modulus of elasticity (42.7 GPa).
- Steel fibers altered the failure mode of concrete by increasing ductility, energy absorption, and post-cracking resistance compared to plain concrete.
- The results showcase the potential of microfibers and nanomaterials to enhance the mechanical properties of concrete. Further research can optimize the proportions and types of micro and nanoscale reinforcements.

The strategic use of steel microfibers and nano-silica integration can lead to stronger, tougher, and more durable concrete, enabling wider implementation in structural and construction applications.

### Supplementary Information

Supplementary Information.

## Data Availability

The data and code associated with this research are accessible to readers. Data can be obtained upon request by contacting Ratchagaraja Dhairiyasamy.

## Abbreviations

W/C: Water to cement ratio
CPV: Cementitious paste volume
MK: Metakaolin
SF: Silica fume
PD: Packing density
WFT: Water film thickness
SFT: Slurry film thickness
FRC: Fiber reinforced concrete
PP: Polypropylene
FF: Fiber factor
FAS: Fiber-aggregate skeletons
UHPC: Ultra-high-performance concrete
ICC: Infilled cementitious composites
VCD: Volume-based concrete mix design
ULCC: Ultra-low cement/carbon concrete
WGP: Waste glass powder
HSFRC: High strength fiber reinforced concrete
RAC: Recycled aggregate concrete
NA: Natural aggregate
RA: Recycled aggregate
UHPFRC: Ultra-high performance fiber reinforced concrete
MRFAIN: Micro concrete reinforced with steel fibers with incorporation of nanomaterials
*A/C*: Effective water/cement weight ratio
*A/L*: Water/whistling weight ratio
*Fcm*: Average value of concrete modulus of elasticity
*F*: Strength
*G.F.*: Fracture energy
*H.R.*: Relative humidity
*I*: Void index
*R*: Linear regression coefficient
*T*: Temperature
*Vf*: Fiber volume
*W*0: Full deformation work
