# Supplementary material for: A comprehensive study on enhancing of the mechanical properties of steel fiber-reinforced concrete through nano-silica integration
Source: Sci Rep. 2023 Nov 16;13:20092. doi: 10.1038/s41598-023-47475-0 (PMC10654428; doi:10.1038/s41598-023-47475-0)
Supplement: Supplementary file 1 — Supplementary Information. [file 41598_2023_47475_MOESM1_ESM.docx]

***Production of mixtures***

The specimens produced are intended to assess the properties of concrete in the hardened state and were made following the NP EN 12390 standards, following all the recommendations in terms of dimensions and respective tolerances. This standard specifies that the test specimens should be cylindrical or cubical, with a minimum size of 100mm in diameter or height. The specimens should be made using fresh concrete, mixed, and handled using a standardized procedure. The concrete mix design and water-cement ratio should be documented, and the specimens should be cured under specific temperature and humidity conditions. For the execution of the kneading of the reference MRFAIN, all the constituents were previously weighed using a digital scale with a precision of one decigram. After weighing, the constituents were introduced into a mixer with a vertical axis, with a capacity of 2 lit (Phase 1) and 100 lit (Phase 2), as seen in Figure 1.


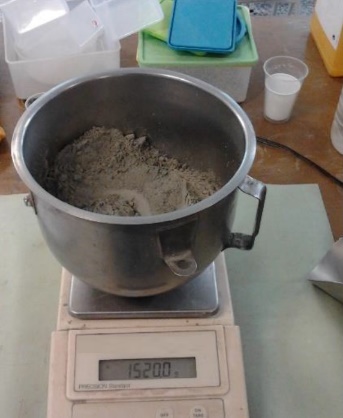


Figure 1 Production of mixtures in phase 1

In concrete with fibers, these were inserted last, and it was found that they dispersed easily during mixing up to a dosage of 2% by volume. After mixing, tests were conducted to characterize the concrete in the fresh state, as already mentioned, and the moulds for the characterization tests were filled in the hardened state. The moulds used to produce the different specimens relating to Phase 1, have the following dimensions: prismatic samples, 40×40×160 mm^3^, for tests.


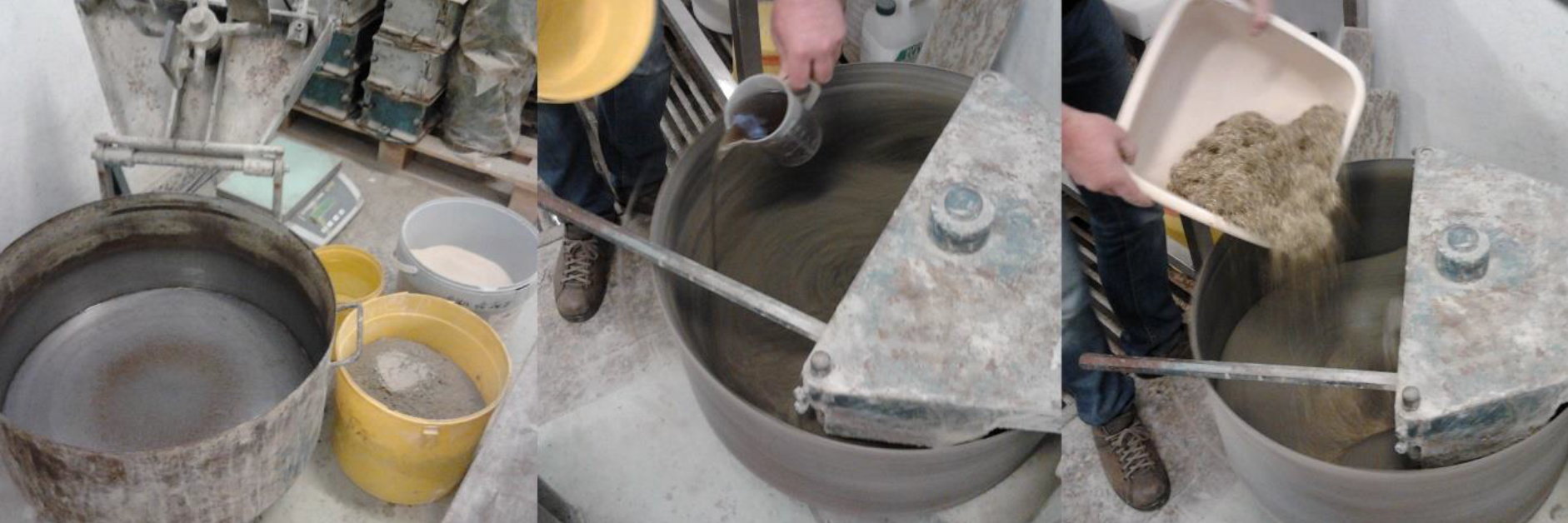


Fig ure 2 Production of mixtures in phase 2

Mix the MRFAIN in Phase 2, all the constituents were previously weighed using a digital scale with a precision of one gram. After weighing, the constituents were introduced into a vertical axis mixer with a capacity of 100 lit, as shown in Figure. 2. In concrete with fibers, these were finally inserted, with the matrix already homogeneous and fluid, and it was verified that they dispersed easily during mixing. After mixing, micro concrete characterization tests were conducted in the fresh state, and the characterization test moulds were filled in the hardened state as in Figure. 3. The moulds used to produce the different specimens have the following dimensions:- 3 cubic specimens, 100 mm on edge, for the compressive strength test; - 2 prismatic specimens, measuring 100×100×400 mm^3^, for the bending test; - 1 prismatic specimen, measuring 100×100×400 mm^3^, for the modulus of elasticity test; - 1 prismatic specimen measuring 100×100×500 mm^3^ in height, for fracture energy testing.


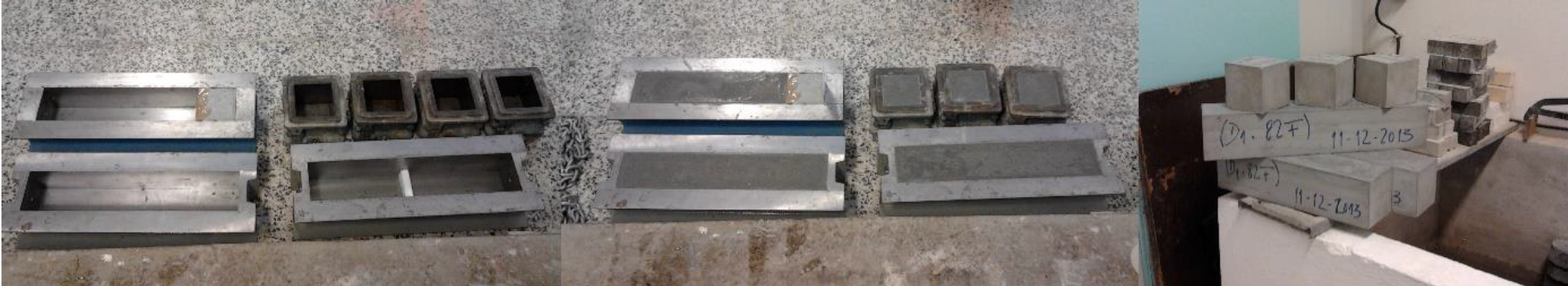


Figure 3 Evolution of the test pieces of phase 2

Two types of curing were studied: normal curing, designated by code N, and special curing with temperature and humidity, designated by code T.

The specimens relating to the Phase 1 mixtures underwent both types of curing. The specimens of the mixtures concerning Phase 2 Final Phase were only subjected to special curing, as it was expected to be the process that would maximize the properties of the concrete.

The specimens, subject to normal curing, were demoulded 24 hours after concreting, subsequently identified, and placed immersed in a water tank at a temperature of 20°C, where there was a resistance with a thermostat, to control the temperature.

The specimens subjected to special curing were placed, after demoulding at 48 hours, inside a thermo-hygrometric chamber, set at a temperature T = 60°C (± 2°C) and a relative humidity RH = 95%.

- 1. *Workability, air content, and density*

The characterization in the fresh state was conducted by spreading tests and determination of the density and air content. The self-compacting mixtures' spreading occurs by gravity's action, measuring its final diameter as in Figure. 4(a) to obtain the fluidity parameter. The density of mortars and concrete can be determined by weighing the volume of the mixture that fills the lower container of the aerometer, with a 1 litre capacity, after releasing the air, without compaction Figure. 4(b) The weighing result corresponds to the apparent density in the fresh state, in kg/dm^3^.

Adjust the composition of the mixtures, it is essential to determine the air content, which is subject to significant variations depending on the constituents and parameters of the binder paste. The variation in the air content of the binding paste causes relevant changes in the mechanical properties and durability [28]. In addition to the H/L ratio and consistency, the target value for air content was kept constant in each run, with slight deviations. For each mixture, the air content was characterized experimentally using the aerometer, which indicates the air content in % Figure.4(c).


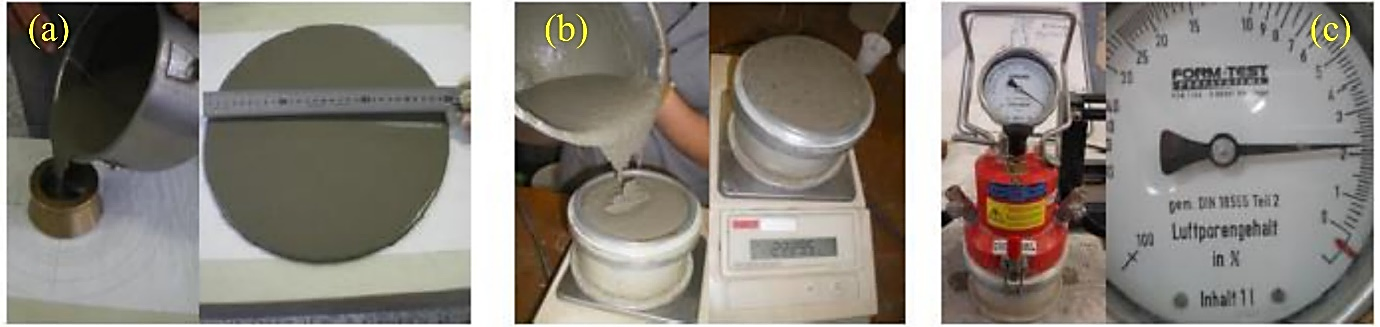


Figure 4 Characterization of self-compacting mortars in the fresh state: (a) spreading; (b) density; (c) air content.

In addition to the characterization in the fresh state, the mixtures were also experimentally characterized in the hardened state. The specimens were produced to evaluate the properties of the concrete in the hardened state and made following the NP EN 12390 standards, following all the recommendations in terms of dimensions and respective tolerances. The standard also specifies acceptance criteria for the compressive strength results obtained from evaluating the specimens. The average compressive strength of three specimens should be considered the concrete's characteristic strength and at least equal to the specified compressive strength for the application. The respective tests also followed the recommendations of the NP EN 12390 and RILEM standards. In the first phase (Phase 1), prismatic specimens measuring 40×40×160mm^3^ were produced and subsequently tested at 28 days in compression and tension. After a preliminary analysis of the results obtained, optimized mixtures were conducted (Phase 2), with the resulting specimens as in Figure. 5 having the dimensions. In addition, the individual strength results should not vary by more than a certain %, as specified in the standard.


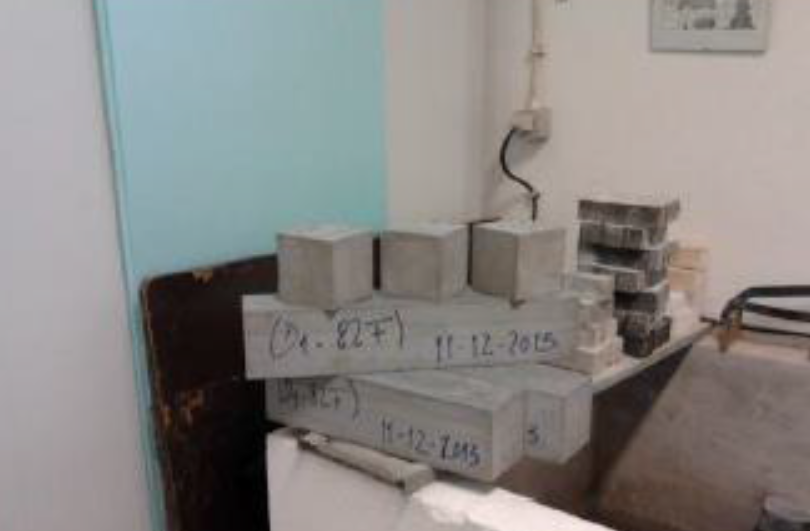


Figure 5 Photographic view of Phase 2 samples.

With this set of tests, it is possible to characterize the behavior of this type of concrete, with the tests in this phase of the study being conducted for 28 days of the age of the concrete.

The mechanisms leading to the results in the study can be inferred from the properties of the materials used and the experimental methodology employed. Adding nanoparticles and steel fibers to the high-strength concrete matrix improved mechanical properties, including higher energy absorption capacity, increased ductility, tensile and compressive strength, and compactness. The % of fibers was carefully considered to ensure compatibility with the mechanical properties of the fibers and matrix. The use of HD smoke silicas, Comital limestone filer, f-type fly ash, Sibelco silica flour, and Silica flour from Sibelco as additives for the concrete matrix was based on the desire to improve concrete performance. Smoke silicas and fly ash were chosen due to their ability to increase mechanical strength and the durability of concrete. Lime filer and silica flour were used to increase the volume of ligating powder without increasing the desired cement act, which fills the voids. The addition of Dramix brand steel micro-fibers with a high carbon content and commercial designation 0.12/10 also contributed to the improved mechanical properties of the concrete matrix. The fibers were chosen to study their influence on the workability of the mixtures and resistance of the specimens, as well as the intended effect of nano-silica on the adhesion between fibers and matrix. The influence of only the addition of nano-SiO_2_ was addressed in the study. The nano-silica used was produced by synthesis by Smart Innovation, based on oxides, and with a minimum purity of 99.5%. Its density obtained in the experimental characterization was 2.22 kg/dm^3^. The mechanisms leading to the results in the study are related to the properties of the materials used and their interactions within the concrete matrix. The study contributes to understanding how additives and fibers can improve the mechanical properties of high-strength concrete matrices suitable for self-compacting concrete.
